# Landscape‐level habitat connectivity of large mammals in Chitwan Annapurna Landscape, Nepal

**DOI:** 10.1002/ece3.70087

**Published:** 2024-08-16

**Authors:** Jagan Nath Adhikari, Bishnu Prasad Bhattarai, Suraj Baral, Tej Bahadur Thapa

**Affiliations:** ^1^ Central Department of Zoology, Institute of Science and Technology Tribhuvan University Kathmandu Nepal; ^2^ Department of Zoology, Birendra Multiple Campus Tribhuvan University Bharatpur Nepal; ^3^ Nepal Zoological Society Kathmandu Nepal; ^4^ Section of Herpetology Leibniz Institute for the Analysis of Biodiversity Change–Museum Koenig Bonn Bonn Germany

**Keywords:** carnivores, Chitwan National Park, fragmentation, habitat patch, least cost path, primates, suitability, ungulates

## Abstract

The populations of many species of large mammals occur in small isolated and fragmented habitat patches in the human‐dominated landscape. Maintenance of habitat connectivity in the fragmented landscapes is important for maintaining a healthy population of large mammal. This study evaluated the landscape patches and their linkages on two carnivores (leopard and Himalayan black bear) and seven prey species (northern red muntjac, chital, sambar, wild pig, Himalayan goral, rhesus macaque, and langur) between Chitwan National Park (CNP) and Annapurna Conservation Area (ACA) by using the least‐cost path (LCP) approach and the Linkage Mapper tool in ArcGIS. A total of 15 habitat patches (average area 26.67 ± 12.70 km^2^) were identified that had more than 50% of the total studied mammals. A weak relation among the habitat patches was found for chital and sambar (Cost‐weighted distance [CWD]: Euclidean distance EucD >100), showed poor connectivity between the habitat patches, while ratio of CWD and EucD was low (i.e., low LCP) between majority of the patches for muntjac, wild pig and leopard hence had potential functional connectivity along the landscape. Similarly, low LCP between the habitat patches located in the mid‐hills was observed for Himalayan goral and Himalayan black bear. Furthermore, the multi‐species connectivity analysis identified the potential structural connectivity between the isolated populations and habitat patches. Therefore, these sites need to be considered connectivity hotspots and be prioritized for the conservation of large mammals in the landscape.

## Graphical abstract

Maintenance of habitat connectivity in the fragmented landscapes is important for maintaining a healthy population of large mammal. The multi‐species connectivity analysis identified the potential functional and structural connectivity between the isolated populations and habitat patches. Therefore, these sites need to be considered hotspots and prioritized for the conservation of large mammals in the landscape.

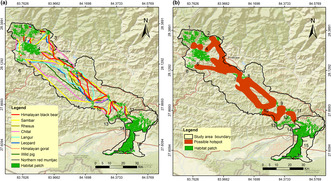

## 1 INTRODUCTION

Landscape functional connectivity refers to the frequency of animal movement between isolated habitat patches, facilitating vital ecological processes such as gene flow, migration, and predator–prey interactions (Ayram et al., 2016; Fahrig, 2003; Fletcher et al., 2018). Poor connectivity increases the extinction risks due to inbreeding depression and limited movement options during adverse conditions (Koen et al., 2014; O'Grady et al., 2006). Therefore, identifying and enhancing landscape connectivity are essential for conservation (Ayram et al., 2016; Koen et al., 2014; Poor et al., 2012; Taylor, 2006). Habitat corridors act as pathways for animal movement between habitat patches, which is essential for maintaining biodiversity (Beier et al., 2008; Howey, 2011; Sarkar et al., 2018) as fragmentation and human activities (e.g., habitat loss, encroachment, developmental activities such as roads, hydropower, urbanization) are restricting animals' movements and landscape connectivity in the globally changing world (Fahrig, 2003; Fletcher et al., 2018; Wilson et al., 2015). However, many studies on the landscape connectivity have targeted a single species, especially, flagship species such as tiger (Dutta et al., 2016; Rathore et al., 2012; Suttidate et al., 2021), Asian elephants (Huang et al., 2019; Vasudev et al., 2021), leopard (Ghoddousi et al., 2020; Kaboodvandpour et al., 2021) for such studies. Generally, Large carnivores are regarded as the habitat generalists and their movement behavior is greatly influenced by the prey availability (del Rio et al., 2001; Lamichhane, 2019; Pokheral & Wegge, 2019) and human disturbance (Bhattarai & Kindlmann, 2013). Maintaining diverse prey species populations in the fragmented landscapes is key for large carnivore conservation. Hence, identification of the habitat patches and potential corridors between them is the best option for the conservation of large carnivores and their prey species (DNPWC, 2016).

A resistance surface is a pixelated map of the landscape in which each pixel has numeric value that indicates the estimated cost of movement through the landscape associated with that pixel (Adriaensen et al., 2003; Unnithan Kumar et al., 2022). Landscape resistance is an important tool for modeling and identifying the potential connectivity along the landscape (Almasieh et al., 2019; Almasieh & Mohammadi, 2023; Carroll et al., 2020). Two approaches have been used to estimate the resistance values – one is expert opinion (Zeller et al., 2012) and field experience; and the other is habitat suitability models (Almasieh et al., 2019, 2023; Ashrafzadeh et al., 2020). Among these approaches, the habitat suitability model is more appropriate to evaluate resistance values and is widely used to model habitat connectivity (Brodie et al., 2015; Koen et al., 2014).

Protected areas (PAs) have played an important role in the biodiversity conservation but PAs are either small or isolated and are unable to hold the viable population of many wild animals (e.g., large carnivores) (Naughton‐Treves et al., 2005; Shrestha et al., 2010). Hence, the forests outside the PAs are also cornerstone for wildlife conservation. This gap can be fulfilled by creating the community–based forest management in the bottlenecks and important corridors (Karanth, Nichols, et al., 2010). Such corridors will also create safe zones for biodiversity, mainly endangered species under climate change and provide an alternative habitat for wildlife (WWF, 2013a, 2013b). Chitwan Annapurna Landscape (CHAL) is one landscape that connects the lowland of Terai with the Himalayas through mid‐hills: highly fragmented and human‐dominated but ecologically diverse geographic region (WWF, 2013a). However, these areas are underrepresented in the protected area network and receive lesser conservation and research priority (Shrestha et al., 2010). Developing corridors between fragmented habitats is one of the best methods for the conservation of animals (Ramiadantsoa et al., 2015; Shrestha et al., 2010), developing a better understanding of their role in biodiversity conservation through horizontal and vertical linkages. The Himalayan Landscape of Nepal still has a large number of natural wildlife habitats, which can be linked through a web of corridors in vertical and horizontal gradients that can increase the chance of long‐term survival of wildlife species by providing better habitats with better shelter, food, and refuge areas. Thus, a landscape‐level study is required to identify corridors between protected areas like CNP and ACA.

Identification of the major environmental variables that influence the habitat suitability, core habitats and connectivity paths is an initial step for the conservation of mammalian species (Eslamlou et al., 2022; Howey, 2011; Latif et al., 2013). Species presence points and environmental variables are the major input of habitat suitability model to predict the suitable habitats as core habitats (Chirima, 2009; Howey, 2011; Sarkar et al., 2018). The least cost path (LCP) is a primary technique for modeling the species distribution through the landscape on the basis of resistance map developed from the habitat suitability map (Dutta et al., 2016; Vasudev et al., 2021). Linkage mapper is a commonly used program to map LCPs that creates a network between the habitat cores and calculates cost‐weighted distances (CWD) and least‐cost paths (McRae & Kavanagh, 2011; Unnithan Kumar & Cushman, 2022), demonstrating its applicability for connectivity studies.

**FIGURE 1:**
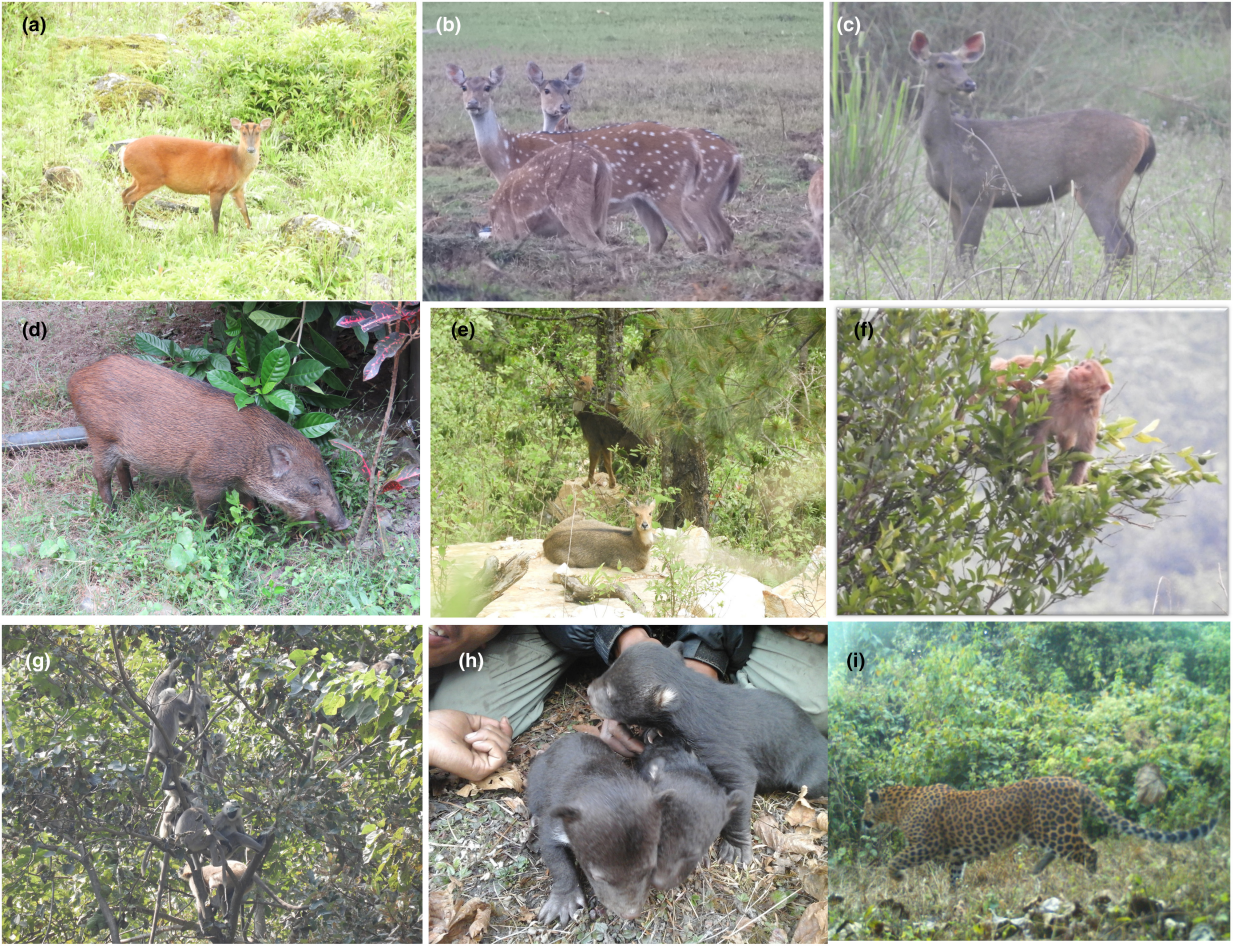
The selected large mammals for modeling (a) northern red muntjac (*Muntiacus vaginalis*), (b) chital (*Axis axis*), (c) sambar (*Rusa unicolor*), (d) wild pig (*Sus scrofa*), (e) Himalayan goral (*Naemorhedus goral*), (f) rhesus macaque (*Macaca mulatta*), (g) langur (*Semnopithecus* spp.), (h) Cubs of Himalayan black bear (*Ursus thibetanus*), (i) leopard (*Panthera pardus*) – reported from camera trap during the study period.

For the modeling of connectivity, we selected two carnivores – leopard (*Panthera pardus* (Linnaeus, 1758)) and Himalayan black bear (*Ursus thibetanus* G. [Baron] Cuvier, 1823); five species of ungulate – northern red muntjac *Muntiacus vaginalis* (Boddaert, 1785), Sambar (*Rusa unicolor* (Kerr, 1792)), chital (*Axis axis* (Erxleben, 1777)), wild pig (*Sus scrofa* Linnaeus, 1758) and Himalayan goral (*Naemorhedus goral* (Hardwicke, 1825)) and two species of primates – rhesus macaque (*Macaca mulatta* (Zimmermann, 1780)) and langur (*Semnopithecus* spp.) (Figure 1). Leopards are widely distributed across Africa and Asia (Stein et al., 2023). The population of the leopards are isolated and dramatically reduced due to habitat fragmentation and loss, human leopard conflict (Adhikari et al., 2024), prey‐based declined from its historic range (Jacobson et al., 2016; Stein et al., 2023), hence, it is listed in vulnerable (VU) category globally (Stein et al., 2023) and nationally (Jnawali et al., 2011). Leopard is the principal predator of Mid‐hill of Nepal that is widely distributed below 4400 m (Jnawali et al., 2011). Himalayan black bear is distributed between the elevation of 1400–4000 m in Nepal and recorded in dense, mixed broad‐leaf forests and steep forest hills (Jnawali et al., 2011). This species is also distributed in Afghanistan, Bangladesh, Bhutan, Cambodia, China, India, Islamic Republic of Iran, Japan, Democratic, People's Republic of Korea, Republic of Korea, Lao PDR, Myanmar, Pakistan, Russian Federation, Taiwan, Province of China, Thailand, Vietnam (Garshelis & Steinmetz, 2020). In Nepal, the estimated suitable area of Himalayan black bear has reduced by 30% within last 10 years (Jnawali et al., 2011). This species is listed in Vulnerable category globally according to IUCN red list (Garshelis & Steinmetz, 2020) and Endangered category as National red list (Jnawali et al., 2011). Habitat fragmentation and loss, poaching for trade in body parts and Human‐wildlife conflict and retaliatory killings are the major threats to this species (Adhikari et al., 2024; Jnawali et al., 2011).

Northern red muntjac is very common and widely distributed species that occurs in Bangladesh, Bhutan, Cambodia, China, Hong Kong, India, Lao People's Democratic Republic, Myanmar, Nepal, Pakistan, Sri Lanka, Thailand and Viet Nam (Timmins et al., 2016). This is listed as least concerned (LC) category globally (Timmins et al., 2016) but listed in vulnerable (VU) category as national red list due to rapid declining of its population within last 15 years in Nepal (Jnawali et al., 2011). There are no official population estimates for this species in Nepal. Chital is the principal prey of tiger and leopard in lowland of Nepal and listed globally as least concerned category (Timmins et al., 2016) and nationally as vulnerable category (Jnawali et al., 2011). Chitals prefer subtropical grasslands and riverine forests (Adhikari et al., 2021). Habitat loss and degradation due to exotic and alien plant species are the major threats to this species (Duckworth et al., 2015; Jnawali et al., 2011). Chitals are the native of Nepal, India and Sri Lanka (Duckworth et al., 2015). Sambar is the largest deer of Nepal and is found in dense Sal and riverine forests of the lowlands and in subtropical forests (Bhattarai & Kindlmann, 2012; Mishra, 1982). The current population of the sambar is estimated as 1200 mature individuals in Nepal (Jnawali et al., 2011). Habitat loss and degradation is the main cause of its declining hence it is listed as vulnerable category both globally (Timmins et al., 2015) and nationally (Jnawali et al., 2011). This species is also the native of Bangladesh, Bhutan, Brunei Darussalam, Cambodia, China, India, Indonesia, Lao PDR, Malaysia, Myanmar, Sri Lanka, Thailand, Viet Nam (Timmins et al., 2015). Himalayan Goral is widely distributed on the forested slopes and steep mountainous areas up to the tree line of Nepal, Bhutan, China (southern Tibet), India and Pakistan (Duckworth & MacKinnon, 2008). These are globally and nationally near threatened species as it is the maximum hunted species (Duckworth & MacKinnon, 2008; Jnawali et al., 2011). Wild pig is highly versatile, widely distributed and often found along the fringes of forests and close to agricultural fields, hence it is also regarded as pest animal and listed as least concerned (Jnawali et al., 2011; Keuling & Leus, 2019).

Rhesus macaque can adapt to any natural habitat and man‐made environment such as buildup areas, human settlements and religious sites (Chalise, 2013; Jnawali et al., 2011). The distribution of the rhesus macaque is common in all places and all types of habitats lower than 3000 m. It is very common and listed as least concerned category (Jnawali et al., 2011; Singh et al., 2020). This is the major crop pest in the mid‐hill of Nepal (Adhikari et al., 2024) and also found in Afghanistan, Bangladesh, Bhutan, China, India, Lao PDR, Myanmar, Pakistan, Thailand, Viet Nam. Hanuman langurs have wide distribution in a range of habitats (Roonwal & Mohnot, 1977). They are distributed in India, Sri Lanka, some parts of Pakistan, most of the areas of Nepal, and some areas in Bangladesh (Karanth, Nichols, et al., 2010; Karanth, Singh, et al., 2010) and are listed as least concerned category (Jnawali et al., 2011; Singh et al., 2020). This species is regarded as pest that causes Human‐wildlife conflict (Adhikari et al., 2024).

Modeling connectivity between patches within a landscape has been identified using single species as well as multispecies but multi‐species modeling is regarded as more effective (Brennan et al., 2020). The single‐species connectivity models overlay or combine to get a multiple species map and help to detect the hotspots and potential paths for connectivity (Brennan et al., 2020; Meyer et al., 2020; Wang et al., 2018). To date, only limited literature exists concerning the application of species distribution modeling to assess species connectivity in Nepal (Neupane et al., 2022; Shrestha & Kindlmann, 2020; Subedi et al., 2021). However, these studies have primarily focused on the connectivity of individual species. Research addressing the connectivity of multiple species remains limited. Consequently, this study aimed to assess potential connectivity by utilizing habitat suitability information for two carnivores (leopard and Himalayan black bear) and seven prey species: five species of wild ungulates (northern red muntjac, chital, sambar, wild pig, Himalayan goral) and two species of primates (rhesus macaque, and langur).

## 2 MATERIALS AND METHODS

### 2.1 Study area

**FIGURE 2:**
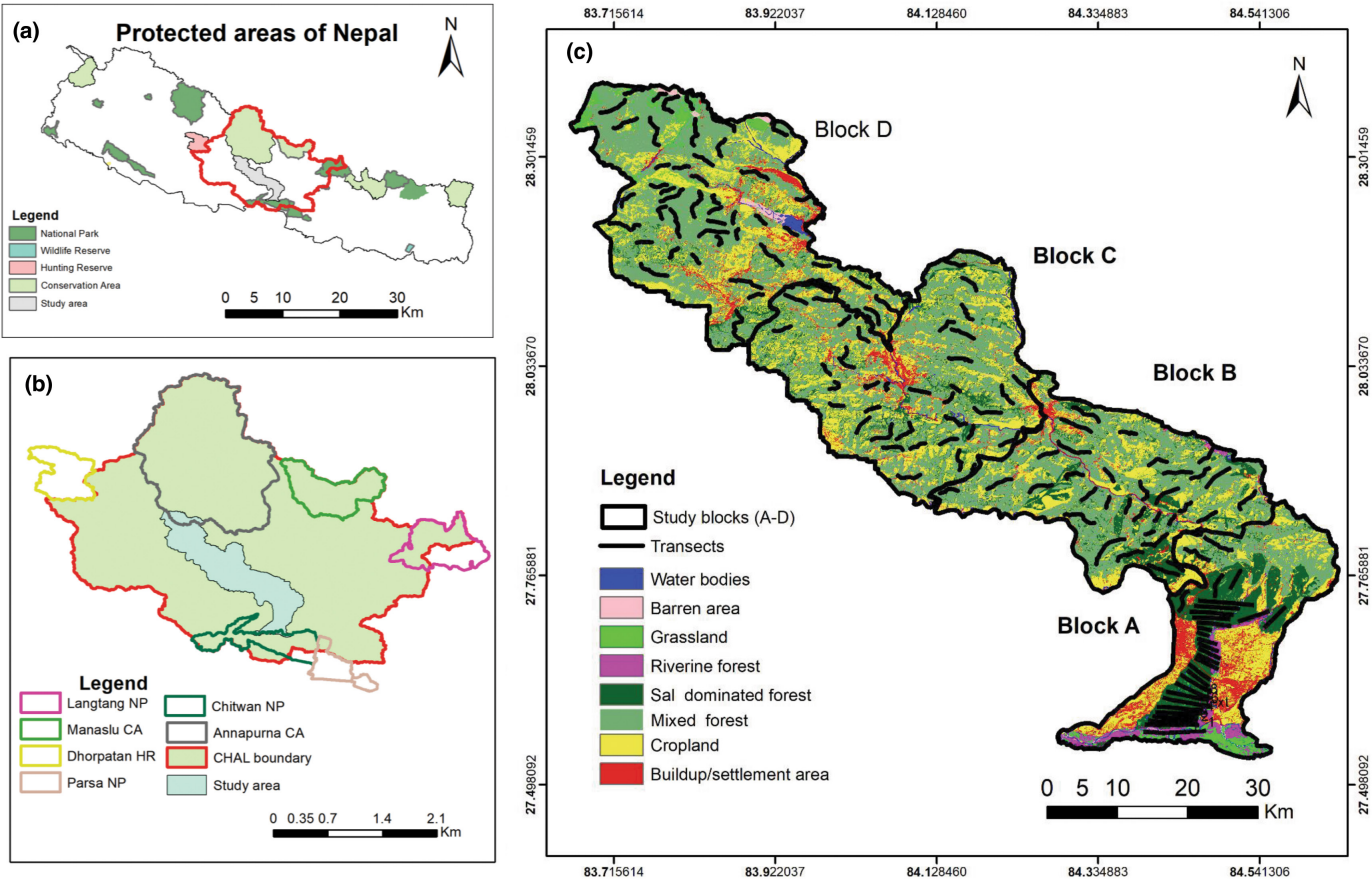
Intensive study area and the inset showing protected areas of Nepal along with study area (a). Protected areas of Nepal showing the study area (b). Chitwan Annapurna Landscape showing the central part (study area) that connects Chitwan National Park in the South with Annapurna Conservation Area in the north (c). Study area showing the blocks A, B, C and D and 150 transects.

The CHAL encompasses entire or partial regions of six protected areas, including Chitwan National Park (CNP) and Annapurna Conservation Area (ACA), and spans across 19 districts in central Nepal. Hydrologically, it is drained by eight major rivers – the Kali Gandaki, the Seti, the Madi, the Marshyandi, the Trishuli, and the Rapti. This study concentrated solely on the central section of CHAL, which serves as a potential link connecting the two protected areas, CNP and ACA. The intensive study area comprises 2749.48 km^2^ and encompasses Chitwan (including the vicinity of Barandabhar Corridor Forest (BCF) and surrounding regions), Tanahun (Seti River basin), Kaski, and certain areas in Syangja and Parbat districts (including Panchase and a portion of ACA). The elevation of the study area ranges from 150 to 3300 m. Due to its significance, this central region has been identified as the top priority corridor for landscape‐level connectivity (WWF, 2013b). The lowland parts of landscape have tropical and subtropical climates, mid‐hills have subtropical and temperate climate, and upper part of mountain has subalpine climate (Paudel et al., 2021). Rich flora and fauna are supported by topography and climate variability (DFRS, 2015) (Figure 2).

This landscape (CHAL) supports three global 846 Ecoregions viz. Terai–duar Savanna and Grasslands, Himalayan Subtropical Broadleaf Forests, Himalayan Sub‐tropical pine forest (Dinerstein et al., 2017; Wikramanayake et al., 2002), and two Ramsar sites: Beeshazari and associated lakes, Chitwan and Lake Clusters of Pokhara valley, Kaski (NLCDC, 2020). CHAL supports a diverse range of mammals, birds, herpetofauna, fishes, micro‐ and macroinvertebrates. (Bhuju et al., 2007; WWF, 2013a).

### 2.2 Methods

#### 2.2.1 Occurrence points collection

The study blocks were divided into four distinct blocks, namely A, B, C, and D considering landscape features, the main courses of rivers, and topographical features (Figure 2c). Block A covers the BCF, part of CNP and surrounding areas of BCF (Kabilas, Jugedi, Kerabari, Chaukidanda, Simaldhap) up to Mahabharat range (it is running closely parallel to the Chure range and separates the Terai with the Hill region, i.e., mid hill) of Chitwan district. Block B covers human‐dominated mid‐hill landscapes such as Devghat, Bandipur, Abu Khairani Rural Municipalities and Vyas Municipality of Tanahun district. It follows the Seti and Trishuli River basin along with mid‐hills. Block C covers the Bhimad Municipality, parts of Rishing Rural Municipality, Ghiring Rural Municipality, Magde Rural Municipality and Shuklagandaki Municipality of Tanahun District and Rupa Rural Municipality of Kaski District along the Seti River basin. Block D covers Bharatpokhari, Nirmalpokhari, Pumdibhumdi, Panchase, Lumle, Ghandruk, Landruk, Deurali and the Australian Camp area. This block harbors four types of forests: national forest, community forest, protected forest (Panchase) and conservation area (Annapurna).

Transects were laid for the collection of presence points of selected species (nine species of mammals) in the landscape (Figure 1). The presence points of ungulates and primates were collected on the basis of direct sighting whereas, the presence points of the carnivores were collected on the basis of signs left by them (e.g., scats, scrapes, pugmarks, scent spray, etc.). Transect size and length were determined based on forest patch size. After identifying forest patches using a topo‐base map (Esri, 2017), transects were overlaid, with patches selected based on diameter; patches less than 2 km in diameter were excluded. Transects (150 out of 164) were systematically laid out according to patch size and accessibility in four blocks (31 in A, 35 in B, 38 in C, and 46 in D). Inaccessible areas (14 transects) due to deep river gorges, steep mountains, and swampy lands were excluded. Transect lengths ranged from 1.18 to 7.84 km, with a minimum 500 m separation in regular forest patches, varying in scattered habitats like Mid hills (Figure 2c; Table S1). We also collected presence of those mammals opportunistically from other possible sites of the study area (e.g., croplands, river banks). These presence coordinates were recorded by using the Global Positioning System (GPS – Garmin eTrex 10). The collected occurrence data were spatially filtered in 30 m by using the Spatially Rarify Occurrence Data tools of SDMtoolbox 2.0.0 in ArcGIS (Brown, 2020; Kaboodvandpour et al., 2021). The filtered data were converted into .CSV format for Maxent modeling (Table 1). The large mammals whose presence locations were <25, were removed from further analysis.

**TABLE 1:** Total presence points collected and spatially rarefied data of the mammals.

SN	Mammal	Code used	Total presence points	Points after spatially rarify (30 m)
1	Leopard	Leopard	289	286
2	Himalayan black bear	Black_bear	49	49
3	Himalayan goral	Goral	54	53
4	Northern red muntjac	Muntjac	265	264
5	Chital	Chital	141	137
6	Wild pig	Wild pig	124	122
7	Sambar	Sambar	26	25
8	Rhesus macaque	Rhesus	212	201
9	Langur monkey	Langur	87	87

#### 2.2.2 Environmental variables

To minimize the risk of over‐fitting the model and develop the most parsimonious model, the environmental variables were selected based on field knowledge, experts' suggestions and extensive literature review of studied large mammals (Dickman & Marker, 2005; Mishra, 1982; Rather et al., 2020; Watts et al., 2019). The slope and terrain ruggedness index (TRI) were extracted by using the digital elevation model (DEM) in ArcGIS 10.8 (ESRI, 2019). The classified image from Landsat (acquisition date 2020‐03‐17) (Landsat 8, Operational Land Imager [OLI]) was used for calculating the Euclidian distances to the nearest forest, grassland, water sources, developed area or human settlements and cropland. We classified the images into eight different classes (Water sources, barren area, grassland, riverine forest, Sal‐dominated forest, mixed forest, cropland and developed area) by using supervised classification based on the ground‐truthing points (Adhikari et al., 2022). Among these classified eight classes, we merged riverine forest, Sal dominated forest and mixed forest as single forest layer. We extracted water sources, grassland, forest, cropland and developed area from the available data and calculated Euclidian distances in ArcGIS 10.8 to be used as environmental variables for modeling. The Normalized Difference Vegetation Index (NDVI) is the most popular and used to quantify the greenness of the vegetation, vegetation density and detect the changes in plant health using red and near infra‐red bands of a remotely sensed image (Pettorelli et al., 2011; USGS, 2022; Yengoh et al., 2015), hence we selected NDVI as one environmental layer for mammals. Additionally, the modified Normalized Difference Water Index (MNDWI) is calculated by using the green and Short‐wave Infrared (SWIR) bands and it enhances the features of open water. MNDWI also minimizes the features of developed areas which are correlated with open water in other indices (Xu, 2006; Xu & Guo, 2014). Furthermore, the Normalized Difference Built‐up Index (NDBI) is a ratio that minimizes the effects of terrain brightness differences and atmospheric effects (Zha et al., 2003). Two spectral bands NIR and SWIR are used to enhance the build‐up or developed area, thus differentiating built‐up over the natural area. The values of each environmental variable were extracted at presence locations (Table 1). For the layer of prey richness of leopard, the suitability map of preys was calibrated as 0 for absent and 1 for the present of the species based on mean equal test sensitivity and specificity logistic threshold. Then, these layers were combined as a single layer.

A total of 13 environmental variables were used for the modeling (Table 2). The variables were differed on the basis of nature of the mammals (Table 2). The selected variable layers were converted into ASCII format with the same resolution, extent and projection system. The spatial resolution of 30 m and UTM 45 N projected coordinate system was used for the modeling.

**TABLE 2:** The environmental variables used in habitat suitability of mammals.

Group	Variables	Predictor code	Description	Units	Source	Selected variables for
Habitat types	Distance to forest	Forest	Euclidean distance to forest	M	Supervised classification of Landsat image 8 (OLI) of 2020	Northern red muntjac, Chital, Sambar, Himalayan goral, wild pig, Rhesus macaques, Langur, Himalayan black bear, leopard
	Distance to grassland	Grass	Euclidean distance to grassland	M		Northern red muntjac, Chital, Sambar, Himalayan goral, wild pig, Rhesus macaques, Langur, Himalayan black bear, leopard
	Distance to water sources	Water sources	Euclidean distance to water sources	M		Northern red muntjac, Chital, Sambar, Himalayan goral, Wild pig, Rhesus macaques, Langur, Himalayan black bear, Leopard
	Habitat heterogeneity	Variety	Total number of habitat variables in 3 × 3 moving window	‐	Classified image of 2020	Northern red muntjac, Chital, Sambar, Himalayan goral, Wild pig, Langur, Himalayan black bear
	NDVI	Ndvi	NIR and Red bands used to calculate NDVI	‐	Downloaded from https://earthexplorer.usgs.gov/	Northern red muntjac, Chital, Sambar, Himalayan goral, Wild pig, Rhesus macaques, Langur macaques, Himalayan black bear, Leopard
	MNDWI	Mndwi	Green and SWIR bands used to calculate MNDWI	‐		Northern red muntjac, Chital, Sambar, Himalayan goral, Wild pig, Langur, Himalayan black bear
Topographic variable	Elevation	Ele	elevation above sea level	M	Digital elevation model	Northern red muntjac, Chital, Sambar, Himalayan goral, Wild pig, Rhesus macaques, Langur, Himalayan black bear
	Slope	Slope	Gradient of slope	‐	Downloaded from https://earthexplorer.usgs.gov/	Northern red muntjac, Chital, Sambar, Himalayan goral, Wild pig, Himalayan black bear, Leopard
	TRI	Tri	Topographic heterogeneity	M		Northern red muntjac, Chital, Himalayan goral, Wild pig, Rhesus macaques, Himalayan black bear, Leopard
Disturbance variable	Distance to cropland	Crop	Euclidean distance to cropland	M	Supervised classification of Landsat image 8 (OLI) of 2020	Northern red muntjac, Chital, Sambar, Himalayan goral, Wild pig, Rhesus macaques, Langur monkeys, Himalayan black bear
	Buildup/settlement area	Dev	Euclidean distance to buildup/settlement area	M		Northern red muntjac, Chital, Himalayan goral, Wild pig, Rhesus macaques, Langur, Himalayan black bear
	NDBI	Ndbi	NIR and SWIR bands are used to calculate build‐up area	‐	Downloaded from https://earthexplorer.usgs.gov/	Northern red muntjac, Chital, Himalayan goral, Wild pig, Langur, Himalayan black bear, Leopard
Richness of prey	Prey species richness of prey	spp_rch	Habitat suitability of preys combined as the single layer	‐	Habitat suitability modeling by using Maxent	Leopard

#### 2.2.3 Habitat suitability models

Maxent develops a model based on series of features (environmental variables) (Phillips et al., 2006). Two types of data (occurrence data and environmental layers) were used for processing in the Maxent program (Phillips et al., 2006). The CSV file of the occurrence points in samples menu and all selected variables layers in ASCII format in the environmental layers' menu bar were loaded for analysis. The replicates and replicated run type were fixed 25 and subsample respectively. The Maxent model ran with 25 iterations and 1000 background points with 70% of the points used as training data and 30% points used as validation of the model. The output of the model was logistic. The performance of the model was evaluated on the basis of AUC values of the receiver operator characteristic (ROC) plot analysis (Phillips, 2008; Phillips et al., 2006; Phillips & Dudík, 2008). The value of the predicted suitability ranges from 0 to 1. The logistic probability of suitability was further regrouped as 0–0.2 = unsuitable, 0.2–0.4 = moderately suitable, 0.4–0.6 = suitable and 0.6–1 = highly suitable (Ansari & Ghoddousi, 2018; Kogo et al., 2019). All the spatial analysis and classification were performed in ArcGIS 10.8 (ESRI, 2019). We used these results of habitat suitability to identify the habitat patches of the species and preparation of resistance layer.

#### 2.2.4 Landscape resistance

The resistance or cost map was prepared using raster habitat suitability map (Figure S1). Every cell on the map has a numeric value that indicates the cost that should be paid to pass through each cell (Bagli et al., 2011; Morovati et al., 2020). The cost map was developed by inverting the value of habitat suitability using the following formula (Almasieh et al., 2019; Morovati et al., 2020).

$$\text{Cost}=100\times \left(1-\text{habitat suitability}\right).$$

The lower cost is assigned to highly suitable areas whereas the highest cost for the habitats with low suitability (Almasieh et al., 2019; Morovati et al., 2020).

#### 2.2.5 Identification of habitat patch

The continuous probability of occurrence was converted to binary predictions of presence and absence based on average equal sensitivity and specificity threshold. The predicted maps of all species were combined to identify the species richness of an area. The habitat patches were defined based on the number of species predicted in that area. About 50% species' present areas with 5000‐pixel size were defined as the patch (Sahraoui et al., 2017).

#### 2.2.6 Modeling connectivity

The LCP algorithms were used to identify a path or corridor (or linkage) between two geographical locations (Adriaensen et al., 2003; Unnithan Kumar & Cushman, 2022). The program Linkage Mapper 2.0.0. (McRae & Kavanagh, 2011) was used to identify the LCP for the movement of mammals from one patch to another. The Linkage Mapper identifies the closer patch, develops the networks between the patches, and calculates the least‐cost distance and paths (McRae & Kavanagh, 2011). The lower cost‐weighted distance is regarded as the strong corridor between two patches. The lower value of LCP is regarded as the lower resistance for the movement of the animals, i.e., has highly suitable (Unnithan Kumar & Cushman, 2022). Two metrics were calculated to show the quality of each linkage. One is the ratio of CWD and Euclidean distance (EucD) that separate each pair of habitat patches. If the ratio of CWD and EucD is equivalent to 1, it is regarded as the highest possible quality linkage (Dutta et al., 2016). The second metric is the ratio of CWD and the length of the LCP. This provides the average resistance encountered along the optimal path between the habitat patches. The least‐cost path of each species was identified and then, combined to find the single multispecies corridor between the patches using raster calculator tools of ArcGIS. The Kernel density estimation method was used to identify the hotspots (Thakali et al., 2015) for the connection of isolated population of mammals in the patches.

## 3 RESULTS

### 3.1 Habitat suitability model

The outcomes derived from the habitat suitability model highlight that several factors play a vital role in predicting mammal occupancy. The performance of habitat suitability models for each mammal was satisfactory (AUC > 0.7) (Table 3). These factors include proximity to cropland, elevation, distance from grassland, forest, water sources, and human settlements, along with the utilization of indices like the NDBI and the NDVI (Table S1). The habitat exhibited varying levels of suitability for different species: 30.29% for the northern red muntjac, 6.45% for chital, 2.6% for sambar, 14.55% for wild pig, 15.55% for Himalayan goral, 34.8% for rhesus macaque, 34.65% for langur, 5.79% for the Himalayan black bear, and 29.94% for the leopard (Table 4).

**TABLE 3:** The habitat suitability model performance using Maxent of the large mammals.

SN	Mammal	AUC	Standard deviation (SD)	Evaluation of predicted model
1	Northern red muntjac	0.737	0.047	Acceptable
2	Chital	0.905	0.023	Outstanding
3	Sambar	0.977	0.007	Outstanding
4	Wild pig	0.794	0.073	Acceptable
5	Himalayan goral	0.809	0.047	Excellent
6	Rhesus macaque	0.725	0.029	Acceptable
7	Langur monkey	0.726	0.033	Acceptable
8	Himalayan black bear	0.832	0.048	Excellent
9	Leopard	0.733	0.014	Acceptable

*Note*: The model was evaluated based on area under curve (AUC) values of the receiver operator characteristic (ROC) plot analysis. The AUC of <0.5 indicates the model did not perform better than random, 0.5–0.6 indicates no discrimination, 0.6–0.7, indicates the discrimination, 0.7–0.8 indicates acceptable, 0.8–0.9 indicates excellent and 0.9–1.0 indicates outstanding (Phillips & Dudík, 2008).

**TABLE 4:** Predicted suitable habitat area for the mammals in CHAL, Nepal (figures in parenthesis indicate the percentage of area).

Group	Logistic probability of suitability	Predicted area (km^2^)								
		Muntjac	Chital	Sambar	Wild pig	Goral	Rhesus	Langur	Black bear	Leopard
Highly suitable	0.6–1.0	179.22 (6.52)	25.76 (0.94)	18.17 (0.66)	160.81 (5.85)	128.01 (4.66)	355.48 (12.94)	223.79 (8.14)	49.77 (1.81)	213.19 (7.75)
Suitable	0.4–0.6	653.49 (23.77)	151.56 (5.51)	53.27 (1.94)	239.23 (8.7)	299.61 (10.89)	598.26 (21.76)	728.92 (26.51)	109.35 (3.98)	610.04 (22.18)
Moderately suitable	0.2–0.4	688.24 (25.03)	58.75 (2.14)	53.16 (1.93)	693.11 (25.21)	581.74 (21.16)	1050.96 (38.22)	1001.24 (36.42)	239.25 (8.7)	826.25 (35.05)
Unsuitable	0–0.2	1228.53 (44.68)	2513.41 (91.41)	2624.88 (95.47)	1656.33 (60.24)	1740.12 (63.29)	744.78 (27.08)	795.53 (28.93)	2351.11 (85.51)	1099.99 (40.00)

### 3.2 Habitat patch

A total of 15 habitat patches were identified in CHAL that supported about 50% or more of the total mammal species predicted (minimum four species). The habitat patches were 26.67 ± 12.70 km^2^ and total patches occupied only 14.56% of total area. The forest in Raipur and Phirphire area was the smallest patch (area = 4.52 km^2^) whereas BCF and surrounding areas were the largest patch (area = 194.36 km^2^) (Table 5).

**TABLE 5:** Location and area of identified habitat patches in landscape.

Patch code	Name	Area (Km^2^)
1	Ghandruk ACA area	44.59
2	Forest, Australian camp area	24.42
3	Lumle	5.79
4	Panchase	59.89
5	Pipaltari to Ramja	6.18
6	Chilaunebas, Bhagera	8.11
7	Raipur, Phirphire area	4.52
8	Tharpek area	5.26
9	Rumsi, Keshabtar area	4.59
10	Bhirkot area	4.59
11	Bandipur area	7.37
12	Ghumaune, Chhimkeshwori area	5.69
13	Kota, Baidi area	15.88
14	Devghat area	8.78
15	Barandabhar and associated area	194.36

### 3.3 Potential corridors

Low resistance areas for the movement of the selected mammals were scattered along the landscape which determined the occupancy of mammals.

**FIGURE 3:**
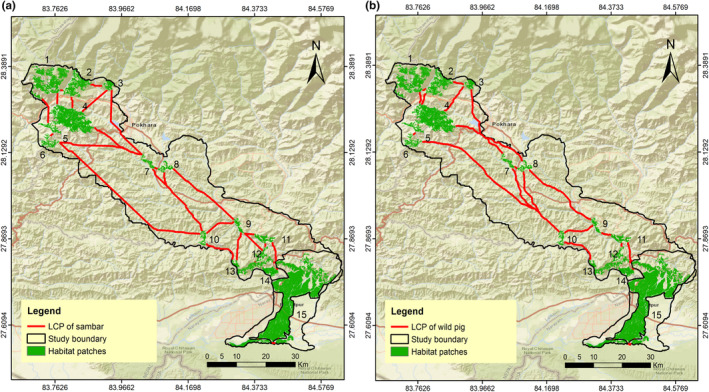
Least‐cost path for (a) sambar, (b) wild pig across major habitat patches in CHAL.

**FIGURE 4:**
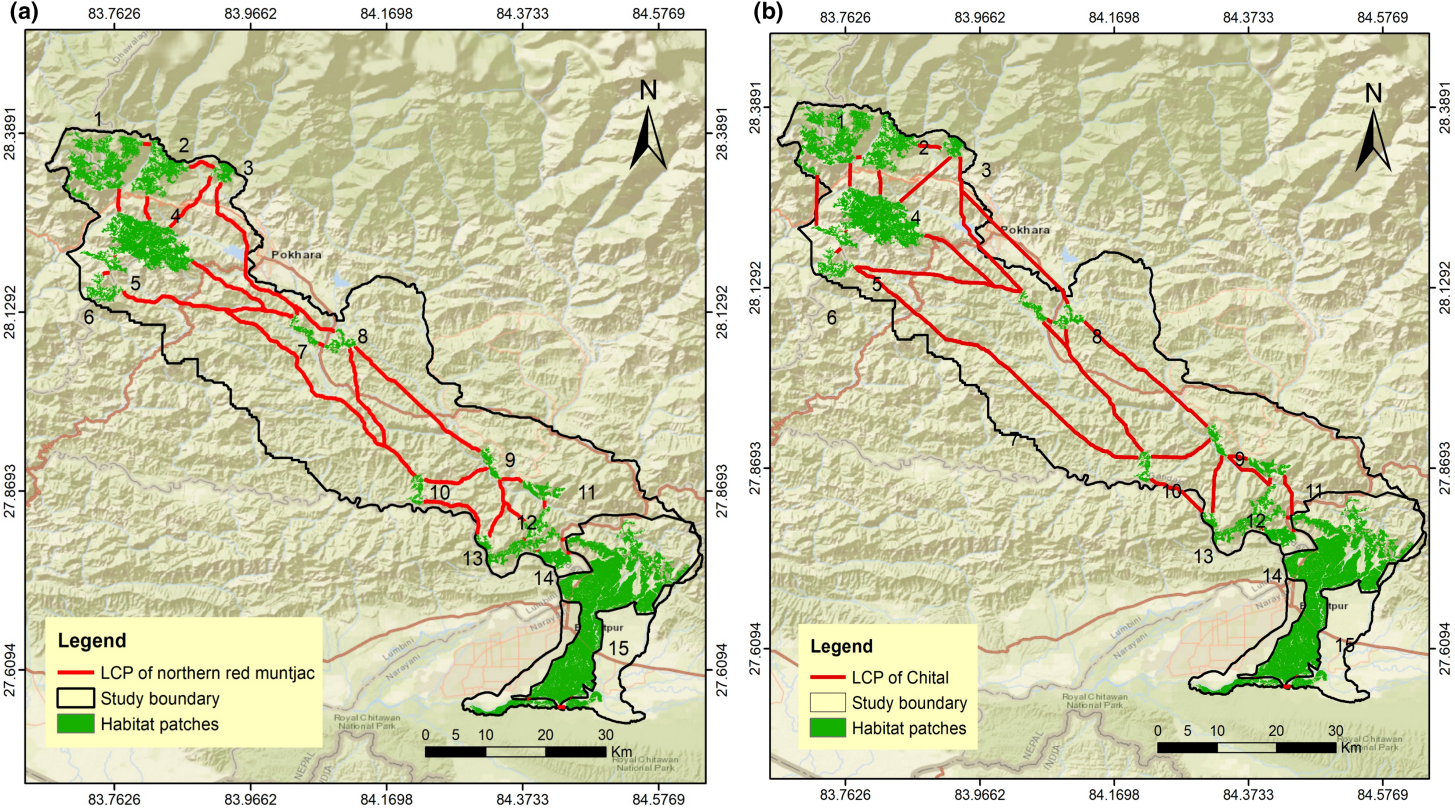
Least‐cost path for (a) northern red muntjac, (b) chital across major habitat patches in CHAL.

**FIGURE 5:**
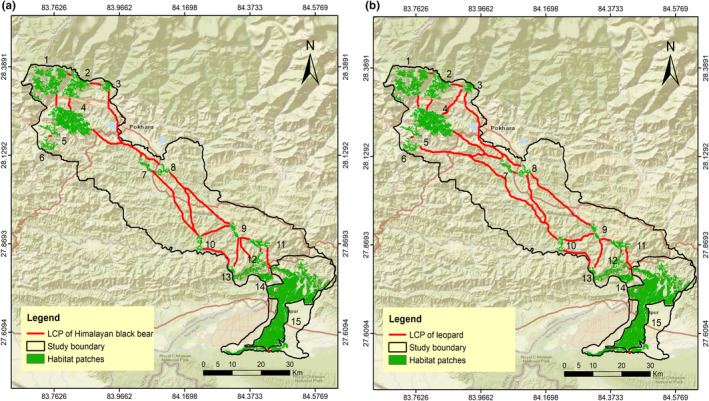
Least‐cost path for (a) Himalayan black bear, (b) leopard across major habitat patches in CHAL.

**FIGURE 6:**
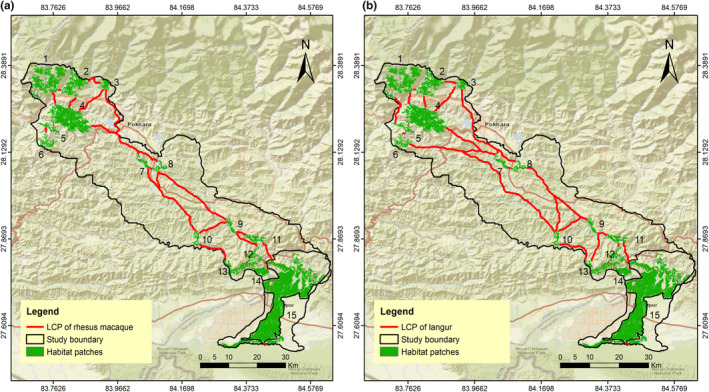
Least‐cost path for (a) rhesus macaques, (b) langue across major habitat patches in CHAL.

**FIGURE 7:**
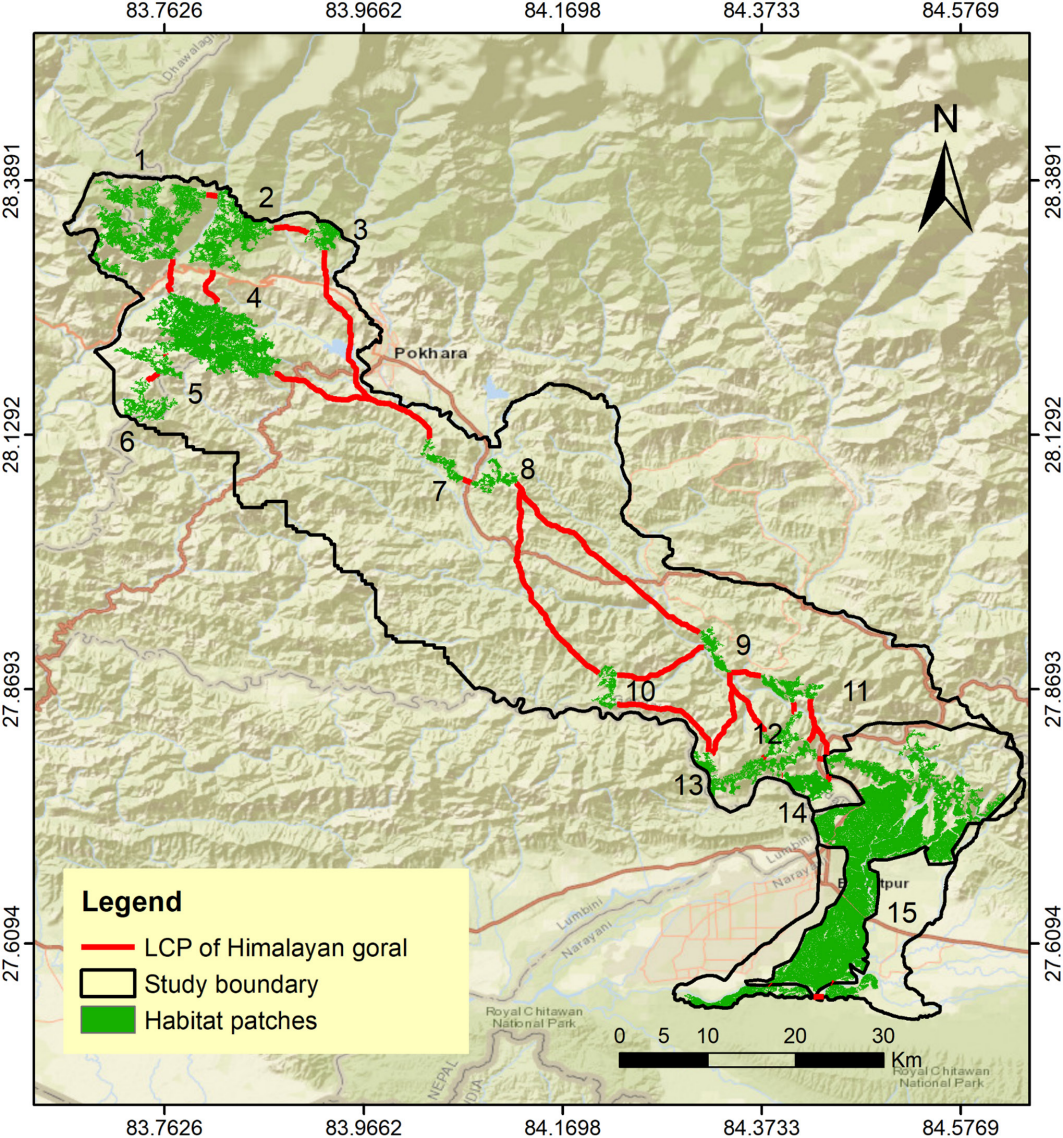
Least‐cost path for Himalayan goral across major habitat patches in CHAL.

**FIGURE 8:**
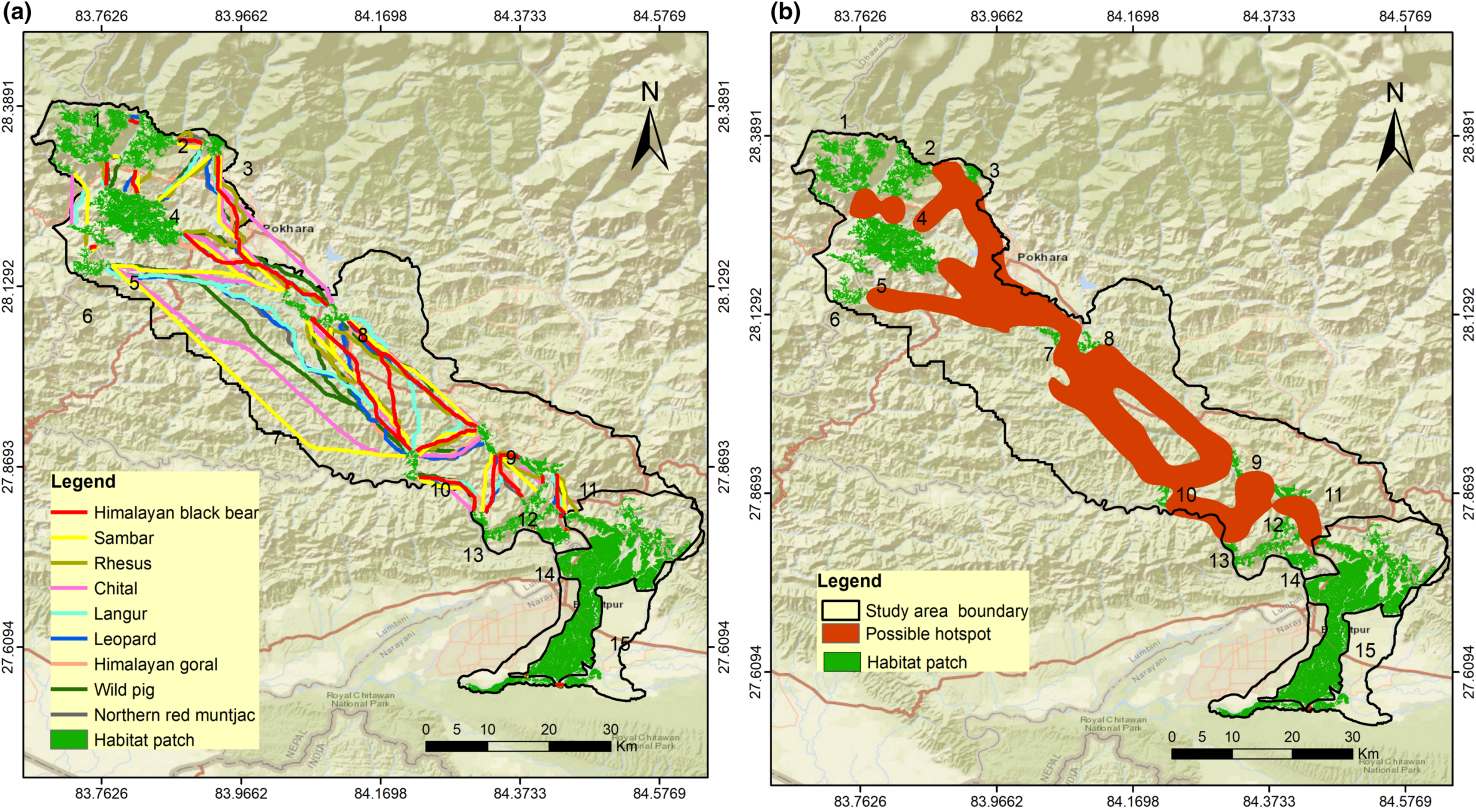
(a) Multispecies connectivity in identified habitat patches of CHAL, (b) Potential areas (hotspot) for the movement of the mammals across major habitat patches in CHAL.

The LCP length of the predicted corridor for multispecies varied from 72 m to 120.63 km. We evaluated 32 LCPs or linkages for sambar (Figure 3a); 31 for Northern red muntjac (Figure 4a), wild pig (Figure 3b), Himalayan black bear (Figure 5a) and leopard (Figure 5b); 30 for chital (Figure 4b), rhesus macaques (Figure 6a) and langur monkey (Figure 6b), and 26 for Himalayan gorals (Figure 7) for linking 15 core habitat patches (Table 6; Tables S2–S10). Among the species, chital and sambar had more resistance and least connected between the habitat patches. Similarly, the LCPs were comparatively higher in the habitat patches of mid hills whereas, the Himalayan black bear showed the strong LCPs among the habitat patches of higher elevation (e.g., Panchase, a lower part of Annapurna Conservation Area). The LCP distance between the habitat patches along the landscape was relatively more appropriate to connect populations of leopard, northern red muntjac, wild pig, rhesus and langur populations than others. The ratio of CWD and EucD was relatively lower in the most of the linkages between the habitat patches. The scattered settlements and major cities such as Vyas, Bhimad, Shuklagandaki and Pokhara were the major resistance to mammals for the connection between patches as the detection probability of these mammals was very low in these cities and settlement areas. The habitat patch in the Rupa, Bagmara to Bharatpokhari and Nirmalpokhari were the major least‐cost path for the mammals as there was maximum detection probability of the mammals (Figure 8a).

**TABLE 6:** Characteristics of linkages of nine mammals.

Mammals	Category	Euclidean distance (EucD, km)	Cost‐weighted distance (CWD, km)	Least‐cost path (LCP, km)	CWD: EucD	CWD: LCP
a. Northern red muntjac	Mean	10.07	689.21	12.56	72.68	59.31
	SE	2.04	143.66	2.64	3.27	1.9
	Range	52.02	3660.55	60.27	121.6	51.32
	Minimum	0.035	4.37	0.072	41.87	30.71
	Maximum	52.05	3664.92	60.34	163.47	82.03
b. Chital	Mean	12.27	1263.11	14.24	106.94	92.86
	SE	2.88	304.09	3.56	3.59	1.83
	Range	83.63	8951.72	107.64	141.59	52.33
	Minimum	0.035	6.94	0.072	65.05	48.12
	Maximum	83.66	8958.66	107.708	206.64	100.45
c. Sambar	Mean	10.07	1065.84	11.43	109.89	95.87
	SE	2.04	217.19	2.38	3.52	1.71
	Range	52.02	5514.71	55.14	128.72	45
	Minimum	0.035	5.8	0.072	78.21	55.59
	Maximum	52.05	5520.51	55.21	206.93	100.59
d. Wild pig	Mean	12.44	940.03	15.23	74.14	60.57
	SE	3.19	251.19	4.34	3.06	2.01
	Range	83.63	6454.13	123.12	80.04	45.61
	Minimum	0.035	3.99	0.072	52.18	37.95
	Maximum	83.66	6458.12	123.19	132.22	83.56
e. Himalayan goral	Mean	12.06	994.67	15.64	82.33	66.55
	SE	2.81	250.2	3.92	3.41	2.97
	Range	83.63	7584.47	120.63	87	64.26
	Minimum	0.035	4.11	0.072	45.6	32.41
	Maximum	83.66	7588.59	120.7	132.6	96.67
f. Rhesus macaque	Mean	10.07	531.946	13.52	61.285	47.35
	SE	2.21	120.49	3.12	4.14	3.67
	Range	52.02	2706.35	76.99	88.49	80.72
	Minimum	0.35	3.47	0.72	24.62	15.11
	Maximum	52.05	2709.82	77.06	113.11	95.83
g. Langur monkey	Mean	10.24	615.03	12.56	71.75	58.91
	SE	2.09	125.3	2.64	3.41	2.16
	Range	52.02	3142.25	60.26	109.79	40.13
	Minimum	0.035	5.45	0.072	45.93	39.48
	Maximum	52.053	3147.69	60.34	155.72	79.61
h. Himalayan black bear	Mean	10.07	980.045	11.91	99.57	83.45
	SE	2.04	208.19	2.45	3.96	2.79
	Range	52.02	5013.4	60.7	138.77	75.11
	Minimum	0.035	6.56	0.072	48.63	25.51
	Maximum	52.053	5019.96	60.77	187.4	100.62
i. Leopard	Mean	10.07	640.58	12.25	69.11	56.87
	SE	2.04	134.55	2.54	2.44	1.94
	Range	52.02	3459.51	60.55	54.8	46.31
	Minimum	0.035	3.69	0.072	50.69	38.04
	Maximum	52.05	3463.19	60.63	105.49	84.35

*Note*: a. Northern red muntjac, b. Chital, c. Sambar, d. Wild pig, e. Himalayan goral, f. Rhesus macaque, g. Langur monkey, h. Himalayan goral and i. Leopard, between the 15 patches in CHAL.

The major hotspots identified the maximum detection probability of mammals and potential LCP for the movement of the isolated populations of the mammals between the patches (Figure 8b).

## 4 DISCUSSION

The successful movement of the animals between the habitat patches in the human‐dominated and fragmented landscape determines the long‐term survival of that animals (Dutta et al., 2016; Thomas, 2000). We found the isolated habitat patches present in this human‐dominated landscape provide the opportunity for connectivity for the isolated populations of the studied mammalian species. This study evaluated landscape‐level single‐species and multispecies connectivity for large mammals (e.g., two carnivores, five ungulates and two primates) across a human‐dominated landscape. We found the habitat patches along the Seti River basin connect CNP with ACA via BCF, forest patches of Tanahun district, Panchase and part of Kaski district. This landscape is the central region of CHAL; hence, the government of Nepal decreased as protective landscape of Nepal and considers a purposed structured corridor (WWF, 2013a, 2013b). From this study, we can say that this landscape is very important for connection of the different scattered population of the mammals and also help for the movement of the large mammals from low land to highland. Similarly, the land use and land cover analysis of 2020 also pointed that more than 62% of the total area of this studied landscape is covered by forest that increases the chances of potential structural corridor between two biologically significant protected area CNP and ACA of Nepal (Adhikari et al., 2022).

Habitat suitability models have provided the basic knowledge on needs and distribution of the species. SDM is very important to identify the potential habitat patches and potential connectivity between them (Dutta et al., 2016; Koen et al., 2014; Phillips & Dudík, 2008). In this study, SDM was used to identify habitat suitability, the habitat patches and investigate the connectivity on the basis of LCP distance between the habitat patches of two carnivores and seven prey species. Basic understanding of the environment variables and their relation with the species is essential for the conservation of species in the landscape (Ahmadi et al., 2017). We modeled the landscape‐level least‐cost path for both single and multi‐species of large mammals based on species distribution modeling. For the least‐cost connectivity, the species distribution model of selected species was used in previous studies (Hanks & Hooten, 2013; Kaboodvandpour et al., 2021; Yu et al., 2015). The least‐cost distance method is a very common, widely used and relatively easy method to model the ecological networks between the habitat patches (Bunn et al., 2000; Sahraoui et al., 2017) though the least‐cost path approaches have some limitations (Dickson et al., 2019; Unnithan Kumar & Cushman, 2022). It is assumed that an animal moves to the particular path as they know the path or corridor very well. But to identify the destination is very difficult for the dispersing animals (Unnithan Kumar & Cushman, 2022). To minimize this limitation, the Kernel density estimation method was used to identify the hotspots (Thakali et al., 2015) for the connection of isolated population of mammals in the patches. Hence, in this study we also used Kernel density methods using the results of LCP to show the possible connectivity path for multispecies. Nowadays, Circuit theory is also used in conservation and it is also a complementary method with other methods (e.g. least‐cost paths) for predicting the movement of animals from one habitat patch to others (Dickson et al., 2019). The results of least‐cost corridor are very important for delivering a clear image of the landscape and serve in the conservation of such probable sites. The results of LCP also help to mitigate the threats to connectivity or suggest restoring it (Ghoddousi et al., 2020). Present study also provided the landscape‐level multi‐species connectivity map to analyze the movement of mammalian species across the human‐dominated landscape and showed the dispersal strength based on the suitability index.

We found 15 different habitat patches along the studies landscape which were connected with each other by different LCPs. The LCPs were varied on the basis of value of LCP distance. The forest patches connect the landscape with two protected areas (e.g., CNP and ACA), but the scattered settlements and cropland become the strong resistance for the connection of isolated populations of the mammals. These forest present in the mid‐hills are fragmented and comparatively smaller in size and can't hold many species of mammals, hence connectivity is required among these habitat patches for the movement of the animals within them. The habitat patches are regarded as undisturbed area with high species richness (Sahraoui et al., 2017). The survival of species in the fragmented landscape depends upon their movement into the different habitat patches (Noss, 1991). The connectivity between habitat patches is important for the species interaction and gene following for the large mammals in the landscape (Borah et al., 2016; Suttidate et al., 2021).

Studies on landscape‐level species connectivity in Nepal are limited. Most of these studies are concentrated on umbrella species (e.g., tiger (Subedi et al., 2021), snow leopard (Shrestha & Kindlmann, 2020)), as it is believed that the associated species would automatically benefit while restoring corridors for a specific species (Carlier & Moran, 2019; Huang et al., 2019; Koen et al., 2014; Shrestha & Kindlmann, 2020). But some range‐specific surrogate species are questionable for their conservation in the corridor (Koen et al., 2014). Hence, this study analyzed the linkages between population of two carnivores and seven prey species of carnivores between the isolated habitat patches. The species which have predicted from different habitat types along the elevation gradients (e.g., leopard) showed low LCP (i.e., strong linkages) between the different habitat patches than ranges specific animals (e.g., chital, sambar). The common species or habitat generalist species such as rhesus and langur monkeys showed strong connectivity to all types of habitat patches, i.e., most of the habitat patches are suitable for monkeys and are less affected by the resistances. Likewise, large habitats of this landscape were predicted as suitable for leopards and showed the strong linkages (i.e., low LCPs) between the habitat patches. The prey species' availability also determines population connectivity and the movement of the predator (Wegge et al., 2009). Leopard is the major carnivore found in this landscape and the LCP distance analysis found that leopards showed the better linkages among the most of the habitat patches as it has a specific home range (6–90 km^2^) (Norton & Henley, 1987; Odden & Wegge, 2005) and has to cover more area for prey. The Himalayan black bear is also the range‐specific carnivore and is commonly found above 1000 m. But sometimes they migrate to the lower elevation even below 1000 m (Bista et al., 2018). The least‐cost analysis indicated its connection towards most of the habitat patches found in mid‐hills and the high hill above 1000 m.

We identified the hotspots for functional connectivity between CNP and ACA as the corridor in the other part of Nepal such as Khata Corridor (connects Bardia National Park, Nepal with Katarniaghat Wildlife Sanctuary) (Gurung et al., 2018), Basanta Corridor (connects Bardia National Park and Sukhlaphata National Park, Nepal with Dudhwa National Park, India) (Gurung et al., 2018), and Laljhadi Maohana Corridor (connects Suklaphanta National Park with Dudhwa National Park, India) (Thapa et al., 2017). Now, these corridors become the model functional corridor in Terai Arc Landscape (TAL) for the movement of large mammals (Gurung et al., 2018). The large cities such as Vyas, Bhimad, Shuklagandaki, Pokhara along with scattered settlements of the mid‐hills are the major resistances to the animal movement. Hence, the forest patches nearer to such areas are very important for connection of the isolated population of mammals. For example: forests of the Rupa to Bagmara, Bharat and Nirmalpokhari areas are very important for connection of population of most of the mammals between habitat patches. Hence, these bottleneck areas must be conserved for maintaining the connectivity between CNP to ACA. Similarly, the forest of the Rumsi and Rishing area nearer to the Vyas are important for animal movement. Bottlenecks are the cornerstones for conservation, and if not properly managed may affect the movement of the animals (Thapa et al., 2018). The identified patches provide critical habitat to existing forest connectivity between CNP and ACA. If conserved well, this corridor will be the model corridor between CNP and ACA.

## 5 CONCLUSIONS

This study identified the structural and functional connectivity for the mammals in the central part of CHAL. Central part of CHAL is the functional corridor (i.e., LCPs) for leopards, northern red muntjac and wild pigs. The range‐restricted mammals such as sambar and chital had higher LCP, that is, had poor connectivity among the habitat patches in the landscape. Likewise, the low LCPs, that is, strong connectivity for Himalayan black bear and Himalayan goral have been identified only in the habitat patches of mid‐hills. The least‐cost paths among the habitat patches, representing connectivity of the different populations of the mammals hold significant importance as habitat and corridors for mammals. Consequently, these findings play a crucial role in assessing the functional connectivity between the two protected areas and offer valuable insights for long‐term conservation efforts. Moreover, these outcomes can serve as a model for other regions within Nepal. Skillful management of these linkages could serve as a fundamental cornerstone in achieving the conservation goals.

## AUTHOR CONTRIBUTIONS

**Jagan Nath Adhikari:** Conceptualization (equal); data curation (equal); formal analysis (equal); investigation (equal); methodology (equal); project administration (equal); resources (equal); validation (equal); visualization (equal); writing – original draft (equal); writing – review and editing (equal). **Bishnu Prasad Bhattarai:** Conceptualization (equal); data curation (equal); formal analysis (equal); methodology (equal); supervision (equal); validation (equal); writing – original draft (equal); writing – review and editing (equal). **Suraj Baral:** Data curation (equal); formal analysis (equal); methodology (equal); writing – review and editing (equal). **Tej Bahadur Thapa:** Conceptualization (equal); formal analysis (equal); methodology (equal); supervision (equal); validation (equal); writing – original draft (equal); writing – review and editing (equal).

## CONFLICT OF INTEREST STATEMENT

The authors declare that there are no conflicts of interest.

## Supporting information


Data S1:

Data S2:

## Data Availability

The presence data used in this study are openly available in Dryad under the DOI: https://doi.org/10.5061/dryad.cnp5hqcb1.
