# Supplementary material for: Landscape‐level habitat connectivity of large mammals in Chitwan Annapurna Landscape, Nepal
Source: Ecol Evol. 2024 Aug 16;14(8):e70087. doi: 10.1002/ece3.70087 (PMC11327774; doi:10.1002/ece3.70087)
Supplement: Supplementary file 1 — Data S1: [file ECE3-14-e70087-s002.docx]

**Supplementary Information 1**

| 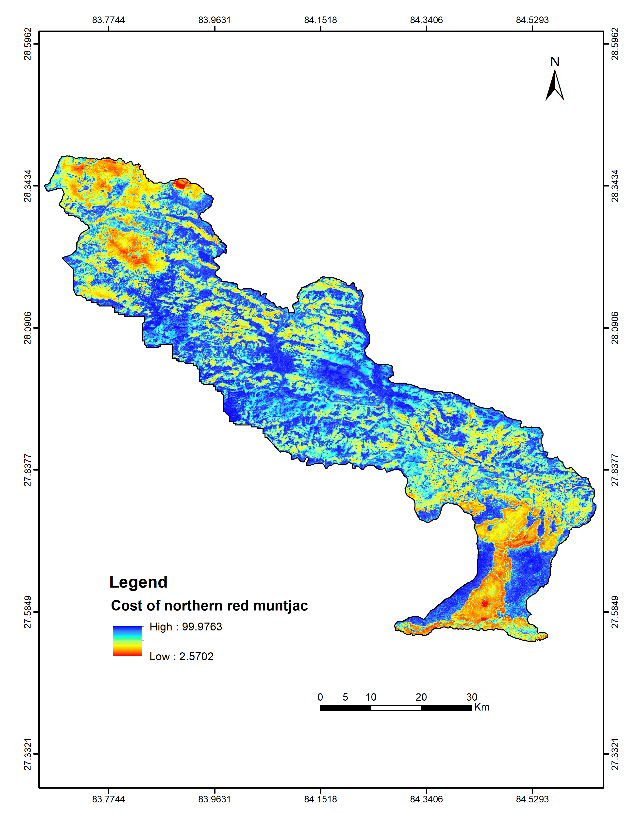 | 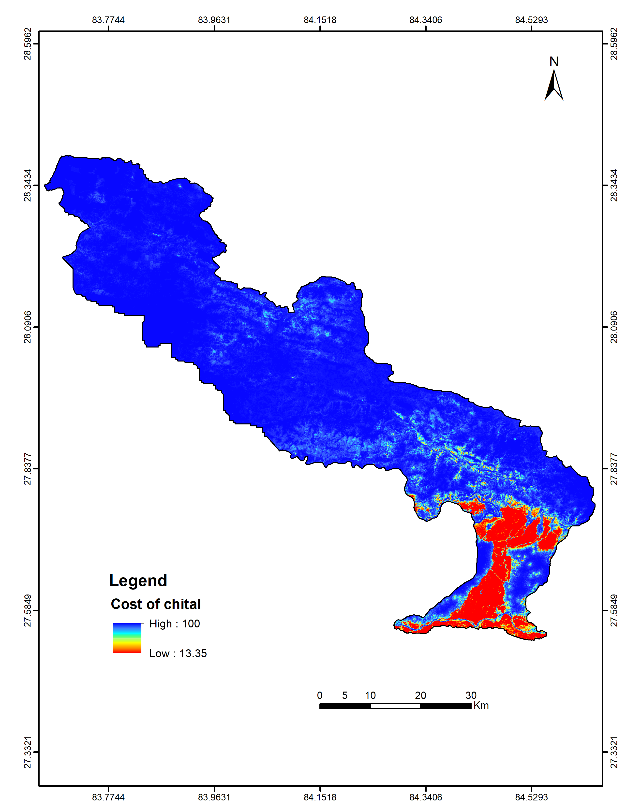 |
| --- | --- |
| Cost map of northern red mutjac | Cost map of chital |
| 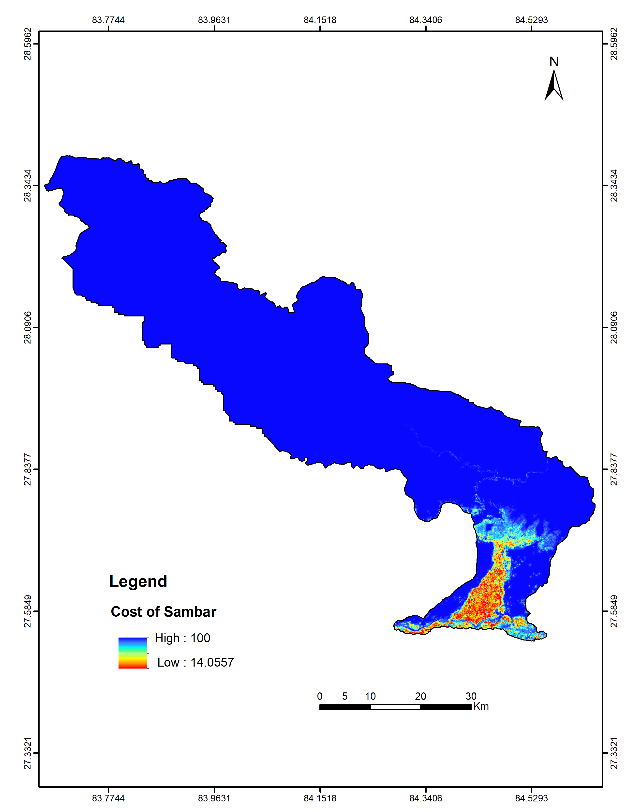 | 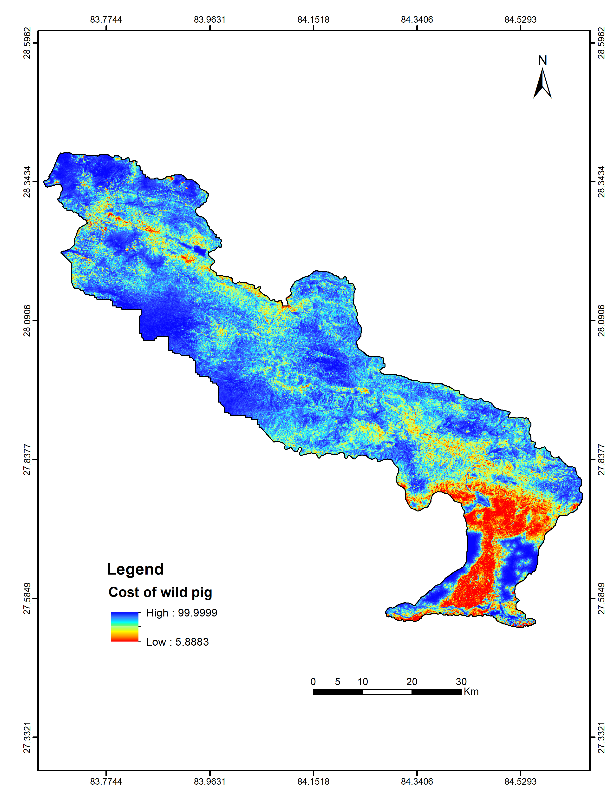 |
| Cost map of sambar | Cost map of wild pig |
| 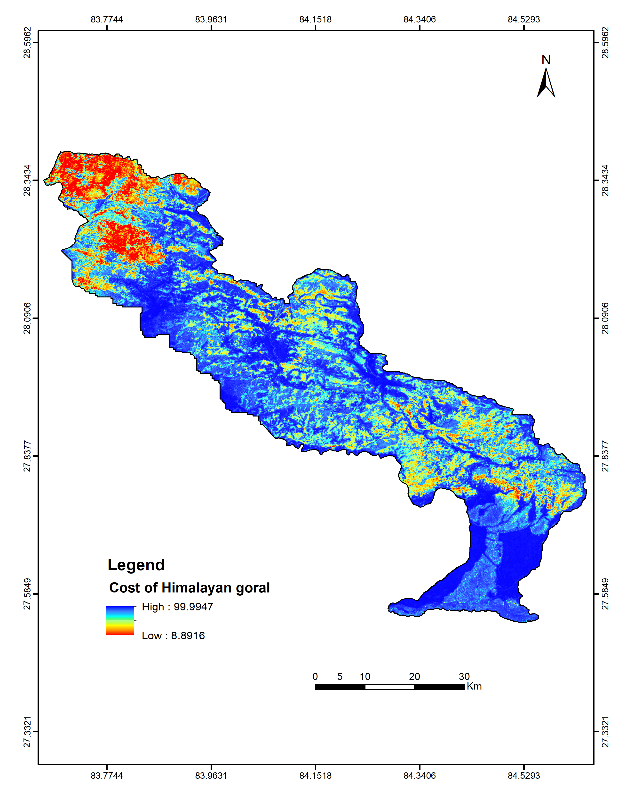 | 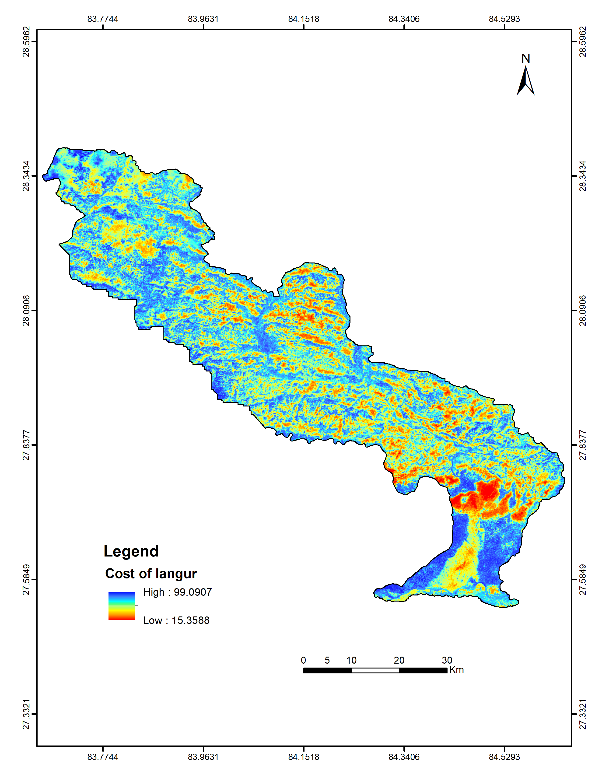 |
| Cost map of Himalayan goral | Cost map of langur |
| 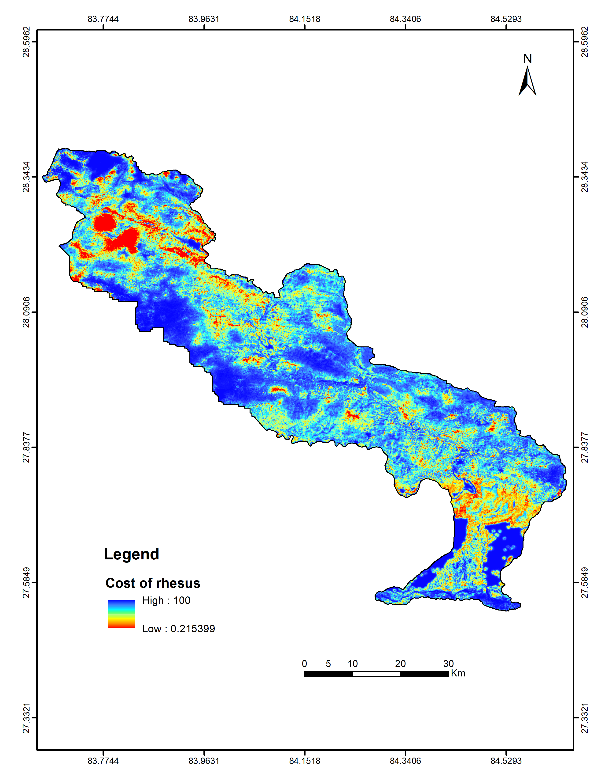 | 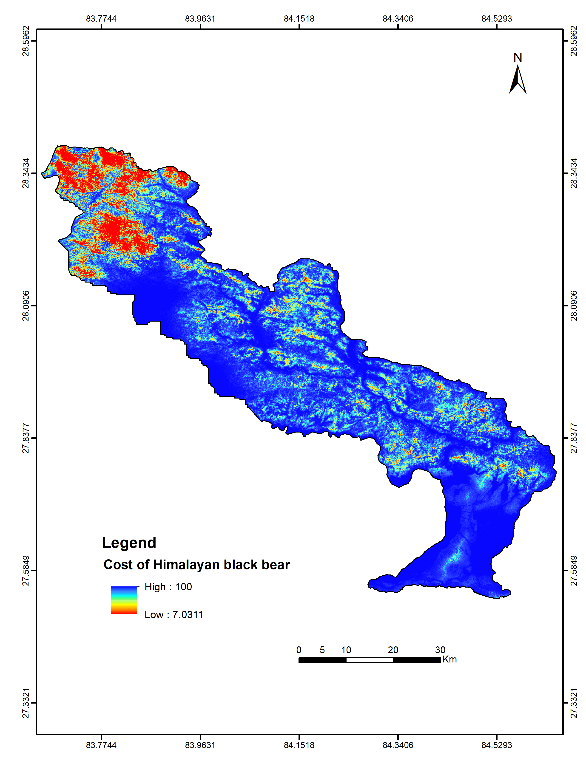 |
| Cost map of rhesus macaque | Cost map of Himalayan black bear |
| 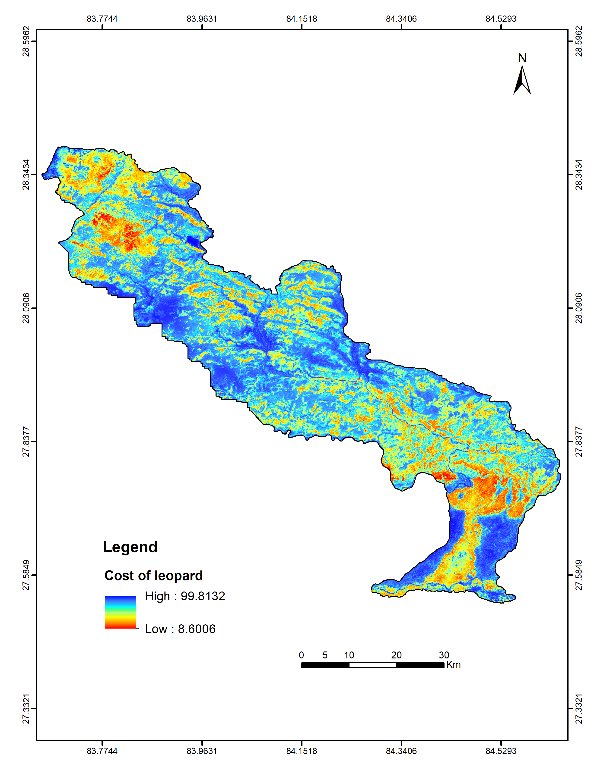 |  |
| Cost map of leopard |  |

**Figure S1.**  Cost or landscape resistance for respective mammals

**Table S1.** Contribution values of environmental variables on habitat suitability. The bold indicates the variable with highest contribution value.

| **Variable** | **Code used** | **Variable contribution (%)** | | | | | | | | |
| --- | --- | --- | --- | --- | --- | --- | --- | --- | --- | --- |
|  |  | **Muntjac** | **Chital** | **Sambar** | **Wild pig** | **Goral** | **Rhesus** | **Langur** | **Bear** | **Leopard** |
| **Distance to cropland** | Crop | **59.3** | 20.4 | 32.4 | 16.5 | **33.1** | 2.5 | **56.3** | 32.6 | 35.1 |
| **NDBI** | Ndbi | 8.5 | 0.6 | - | 3.2 | 2.3 | - | 22.8 | 5.1 | - |
| **NDVI** | Ndvi | 8.1 | 7.5 | 1.3 | 10.5 | 7.4 | 5.9 | 2 | 1.4 | 3.7 |
| **Distance to buildup/settlements area** | Dev | 7.1 | 1 | - | 4.2 | 14.1 | **22.9** | 1.6 | 1 | 6.7 |
| **Distance to grassland** | Grass | 6.8 | 2.2 | 6.7 | 8.8 | 4.3 | 9.8 | 3.5 | 7.4 | 3.5 |
| **Elevation** | Ele | 2.9 | **61.6** | **51.7** | **35.1** | 11.7 | 15.7 | 10.1 | **39.1** | 1.2 |
| **Slope** | Slope | 2 | 1.4 | 1.5 | 5.6 | 0.3 | 4.4 | - | 2.9 | 3.2 |
| **Distance to forest** | Forest | 1.7 | 2.5 | 3.2 | 8.6 | 0.1 | 13.8 | 1.8 | 0.1 | 3.9 |
| **Distance to water bodies** | Water | 1.1 | 0.4 | 0.7 | 1.4 | 11.3 | 17.8 | 0.4 | 7.3 | 2.4 |
| **Index of habitat heterogeneity** | Variety | 1.1 | 0.6 | 0.8 | 1.5 | 0.2 | 2.9 | - | 0.4 | 1.3 |
| **TRI** | Tri | 1 | 1.1 | - | 0.4 | 15.1 | 4.2 | - | 0.4 | 1.8 |
| **MNDWI** | Mndwi | 0.4 | 0.6 | 1.6 | 4.2 | 0.1 | - | 1.5 | 2.3 | - |
| **Prey species richness** | spp_rch | - | - | - | - | - | - | - | - | **37.2** |

**Table S2.** Characteristics of the mapped linkages between the 15 core areas for northern red muntjac in the CHAL. Here, lcDist= Least-cost distance, lcpLength= Least-cost path length, eucDist= Euclidean distance, cwd= cost weight distance

| Link | Core1 | Core 2 | linkType | eucDist | lcDist | lcpLength | *cwd:EucD* | *cwd:lcp* |  |
| --- | --- | --- | --- | --- | --- | --- | --- | --- | --- |
| 1 | 1 | 2 | 1 | 783 | 49745.3 | 1152 | 63.53 | 43.18 | |
| 2 | 1 | 4 | 1 | 3680 | 244219.1 | 3789 | 66.36 | 64.45 | |
| 3 | 1 | 5 | -15 | 8061 | 559286.4 | 10562 | 69.38 | 52.95 | |
| 4 | 2 | 3 | 1 | 3300 | 138180.9 | 4500 | 41.87 | 30.71 | |
| 5 | 2 | 4 | 1 | 3616 | 208790.1 | 4075 | 57.74 | 51.24 | |
| 6 | 3 | 4 | 1 | 9983 | 719282.8 | 11085 | 72.05 | 64.89 | |
| 7 | 3 | 7 | 1 | 23270 | 1682717 | 28809 | 72.31 | 58.41 | |
| 8 | 3 | 8 | 1 | 28402 | 2009464 | 35181 | 70.75 | 57.12 | |
| 9 | 4 | 5 | 1 | 91 | 7757.506 | 144 | 85.25 | 53.87 | |
| 10 | 4 | 6 | -15 | 3401 | 201671.8 | 3772 | 59.3 | 53.47 | |
| 11 | 4 | 7 | 1 | 16885 | 1024916 | 18888 | 60.7 | 54.26 | |
| 12 | 5 | 6 | 1 | 973 | 78463.3 | 1131 | 80.64 | 69.38 | |
| 13 | 6 | 7 | 1 | 24921 | 1591726 | 27864 | 63.87 | 57.12 | |
| 14 | 6 | 10 | 1 | 52053 | 3664921 | 60065 | 70.41 | 61.02 | |
| 15 | 7 | 8 | 1 | 837 | 72216.19 | 929 | 86.28 | 77.74 | |
| 16 | 7 | 10 | -15 | 25444 | 1864858 | 28181 | 73.29 | 66.17 | |
| 17 | 8 | 9 | 1 | 24813 | 1775622 | 26081 | 71.56 | 68.08 | |
| 18 | 8 | 10 | 1 | 22373 | 1659908 | 24838 | 74.19 | 66.83 | |
| 19 | 9 | 10 | 1 | 9146 | 572666.8 | 10705 | 62.61 | 53.5 | |
| 20 | 9 | 11 | 1 | 2949 | 178521.3 | 3181 | 60.54 | 56.12 | |
| 21 | 9 | 12 | 1 | 7500 | 453087 | 8602 | 60.41 | 52.67 | |
| 22 | 9 | 13 | 1 | 9171 | 557745.5 | 10450 | 60.82 | 53.37 | |
| 23 | 10 | 15 | 1 | 9293 | 790274.3 | 12284 | 85.04 | 64.33 | |
| 24 | 10 | 13 | -15 | 33159 | 2376085 | 50016 | 71.66 | 47.51 | |
| 25 | 11 | 12 | 1 | 1020 | 89168.17 | 1087 | 87.42 | 82.03 | |
| 26 | 11 | 15 | -15 | 6291 | 443928.2 | 8134 | 70.57 | 54.58 | |
| 27 | 11 | 13 | -15 | 4423 | 293758.6 | 5234 | 66.42 | 56.13 | |
| 28 | 12 | 15 | -15 | 2877 | 170307 | 3300 | 59.2 | 51.61 | |
| 29 | 12 | 13 | 1 | 35 | 5721.434 | 72 | 163.47 | 79.46 | |
| 30 | 13 | 15 | 1 | 544 | 47755.43 | 637 | 87.79 | 74.97 | |
| 31 | 14 | 15 | 1 | 372 | 33559.39 | 414 | 90.21 | 81.06 | |

**Table S3.** Characteristics of the mapped linkages between the 15 core areas for chital in the CHAL. Here, lcDist= Least-cost distance, lcpLength= Least-cost path length, eucDist= Euclidean distance, cwd= cost weight distance

| Link | Core1 | Core 2 | linkType | eucDist | lcDist | lcpLength | *cwd:EucD* | *cwd:LCP* |
| --- | --- | --- | --- | --- | --- | --- | --- | --- |
| 1 | 1 | 2 | 1 | 783 | 83271.05 | 834 | 106.35 | 99.84 |
| 2 | 1 | 4 | 1 | 3680 | 367531.7 | 3690 | 119.45 | 99.6 |
| 3 | 1 | 5 | 1 | 8061 | 889016.9 | 8985 | 110.29 | 98.94 |
| 4 | 2 | 3 | 1 | 3300 | 350801 | 3521 | 106.3 | 99.63 |
| 5 | 2 | 4 | 1 | 3616 | 382352.3 | 3826 | 105.74 | 99.93 |
| 6 | 3 | 4 | 1 | 9983 | 1032652 | 10352 | 103.44 | 99.75 |
| 7 | 3 | 7 | 1 | 23270 | 2524168 | 25406 | 108.47 | 99.35 |
| 8 | 3 | 8 | -15 | 28402 | 3171151 | 32027 | 111.65 | 99.01 |
| 9 | 4 | 5 | 1 | 91 | 14418.01 | 144 | 158.44 | 100.13 |
| 10 | 4 | 6 | -15 | 3401 | 359142.7 | 3601 | 105.6 | 99.73 |
| 11 | 4 | 7 | 1 | 16885 | 1782723 | 18248 | 105.58 | 97.69 |
| 12 | 5 | 6 | 1 | 973 | 103551.3 | 1035 | 106.42 | 100.04 |
| 13 | 6 | 7 | 1 | 24921 | 2602299 | 26339 | 104.42 | 98.8 |
| 14 | 6 | 10 | 1 | 52053 | 5437384 | 55210 | 104.46 | 98.48 |
| 15 | 7 | 8 | 1 | 837 | 91815.23 | 923 | 109.7 | 99.47 |
| 16 | 7 | 10 | 1 | 25444 | 2647620 | 26679 | 104.06 | 99.23 |
| 17 | 8 | 9 | 1 | 24813 | 2503366 | 25531 | 100.89 | 98.05 |
| 18 | 8 | 10 | 1 | 22373 | 2392380 | 24199 | 106.93 | 98.86 |
| 19 | 9 | 10 | 1 | 9146 | 937348.9 | 9942 | 102.49 | 94.28 |
| 20 | 9 | 11 | 1 | 2849 | 290865.2 | 3106 | 102.09 | 93.64 |
| 21 | 9 | 12 | 1 | 6500 | 719987.6 | 8136 | 110.77 | 88.49 |
| 22 | 9 | 14 | 1 | 8971 | 898017.9 | 9694 | 100.1 | 92.63 |
| 23 | 10 | 14 | 1 | 9293 | 1118833 | 11547 | 120.34 | 96.89 |
| 24 | 11 | 12 | 1 | 1010 | 101982 | 1087 | 100.97 | 93.82 |
| 25 | 11 | 13 | 1 | 5891 | 606283.1 | 6900 | 102.91 | 87.86 |
| 26 | 12 | 13 | -15 | 2377 | 270560.8 | 3148 | 113.82 | 85.94 |
| 27 | 12 | 14 | 1 | 35 | 7232.443 | 72 | 206.64 | 100.45 |
| 28 | 13 | 14 | 1 | 544 | 58841.11 | 600 | 108.16 | 98.06 |
| 29 | 13 | 15 | 1 | 372 | 39421.8 | 414 | 105.97 | 95.22 |
| 30 | 14 | 15 | 1 | 110 | 9212.424 | 132 | 83.74 | 69.79 |

**Table S4.** Characteristics of the mapped linkages between the 15 core areas for sambar in the CHAL. Here, lcDist= Least-cost distance, lcpLength= Least-cost path length, eucDist= Euclidean distance, cwd= cost weight distance

| Link | Core1 | Core 2 | linkType | eucDist | lcDist | lcpLength | *cwd:EucD* | *cwd:LCP* |
| --- | --- | --- | --- | --- | --- | --- | --- | --- |
| 1 | 1 | 2 | 1 | 783 | 83485.28 | 834 | 106.62 | 100.1 |
| 2 | 1 | 4 | 1 | 3680 | 369000 | 3690 | 100.27 | 100 |
| 3 | 1 | 5 | 1 | 8061 | 898528.9 | 8985 | 111.47 | 100 |
| 4 | 2 | 3 | 1 | 3300 | 352154.3 | 3521 | 106.71 | 100.02 |
| 5 | 2 | 4 | 1 | 3616 | 382669.1 | 3826 | 105.83 | 100.02 |
| 6 | 3 | 4 | 1 | 9983 | 1035204 | 10352 | 103.7 | 100 |
| 7 | 3 | 7 | 1 | 23270 | 2540674 | 25406 | 109.18 | 100 |
| 8 | 3 | 8 | -15 | 28402 | 3202727 | 32027 | 112.76 | 100 |
| 9 | 4 | 5 | 1 | 91 | 14485.28 | 144 | 159.18 | 100.59 |
| 10 | 4 | 6 | -15 | 3401 | 360198.1 | 3601 | 105.91 | 100.03 |
| 11 | 4 | 7 | 1 | 16885 | 1824870 | 18248 | 108.08 | 100 |
| 12 | 5 | 6 | 1 | 973 | 103580.7 | 1035 | 106.46 | 100.08 |
| 13 | 6 | 7 | 1 | 24921 | 2633923 | 26339 | 105.69 | 100 |
| 14 | 6 | 10 | 1 | 52053 | 5520509 | 55210 | 106.06 | 99.99 |
| 15 | 7 | 8 | 1 | 837 | 92390.96 | 923 | 110.38 | 100.1 |
| 16 | 7 | 10 | 1 | 25444 | 2667547 | 26679 | 104.84 | 99.99 |
| 17 | 8 | 9 | 1 | 24813 | 2552332 | 25531 | 102.86 | 99.97 |
| 18 | 8 | 10 | 1 | 22373 | 2418407 | 24188 | 108.09 | 99.98 |
| 19 | 9 | 10 | 1 | 9146 | 989634.1 | 9904 | 108.2 | 99.92 |
| 20 | 9 | 11 | 1 | 2949 | 309340.8 | 3094 | 104.9 | 99.98 |
| 21 | 9 | 12 | 1 | 7500 | 799135.9 | 8006 | 106.55 | 99.82 |
| 22 | 9 | 14 | 1 | 9171 | 965044.1 | 9669 | 105.23 | 99.81 |
| 23 | 10 | 14 | 1 | 9293 | 1150222 | 11547 | 123.77 | 99.61 |
| 24 | 10 | 17 | -15 | 33159 | 3654994 | 50247 | 110.23 | 72.74 |
| 25 | 11 | 12 | 1 | 1020 | 107842.3 | 1087 | 105.73 | 99.21 |
| 26 | 11 | 13 | 1 | 6291 | 659900.9 | 6764 | 104.9 | 97.56 |
| 27 | 11 | 14 | -15 | 4423 | 471877.3 | 4762 | 106.69 | 99.09 |
| 28 | 12 | 13 | -15 | 2877 | 293376.8 | 2990 | 101.97 | 98.12 |
| 29 | 12 | 14 | 1 | 35 | 7242.641 | 72 | 206.93 | 100.59 |
| 30 | 13 | 14 | 1 | 544 | 59574.11 | 600 | 109.51 | 99.29 |
| 31 | 13 | 15 | 1 | 372 | 39645.13 | 414 | 106.57 | 95.76 |
| 32 | 14 | 15 | -15 | 17610 | 1790170 | 30406 | 101.6565 | 55.59 |

**Table S5.** Characteristics of the mapped linkages between the 15 core areas for wild pig in the CHAL. Here, lcDist= Least-cost distance, lcpLength= Least-cost path length, eucDist= Euclidean distance, cwd= cost weight distance

| Link | Core1 | Core 2 | linkType | eucDist | lcDist | lcpLength | *cwd:EucD* | *cwd:LCP* |
| --- | --- | --- | --- | --- | --- | --- | --- | --- |
| 1 | 1 | 2 | 1 | 783 | 46780.63 | 1128 | 59.75 | 41.47 |
| 2 | 1 | 4 | 1 | 3680 | 227517.4 | 4137 | 61.83 | 55 |
| 3 | 1 | 5 | -15 | 8061 | 584279.5 | 9959 | 72.48 | 58.67 |
| 4 | 2 | 3 | 1 | 3300 | 281729.5 | 3575 | 85.37 | 78.81 |
| 5 | 2 | 4 | 1 | 3616 | 222801.3 | 4206 | 61.62 | 52.97 |
| 6 | 3 | 4 | 1 | 9983 | 724787.3 | 10844 | 72.6 | 66.84 |
| 7 | 3 | 7 | 1 | 23270 | 1692323 | 26860 | 72.73 | 63.01 |
| 8 | 3 | 8 | 1 | 28402 | 2020895 | 34332 | 71.15 | 58.86 |
| 9 | 4 | 5 | 1 | 91 | 12032.34 | 144 | 132.22 | 83.56 |
| 10 | 4 | 6 | -15 | 3401 | 250124.8 | 3935 | 73.54 | 63.56 |
| 11 | 4 | 7 | 1 | 16885 | 1009733 | 19577 | 59.8 | 51.58 |
| 12 | 5 | 6 | 1 | 973 | 84138.99 | 1070 | 86.47 | 78.63 |
| 13 | 6 | 7 | -15 | 24921 | 1952614 | 32654 | 78.35 | 59.8 |
| 14 | 6 | 10 | 1 | 52053 | 4257815 | 56557 | 81.8 | 75.28 |
| 15 | 6 | 17 | -15 | 83664 | 6458121 | 123195 | 77.19 | 52.42 |
| 16 | 7 | 8 | 1 | 837 | 61151.55 | 996 | 73.06 | 61.4 |
| 17 | 7 | 10 | 1 | 25444 | 1959046 | 28025 | 76.99 | 69.9 |
| 18 | 8 | 9 | 1 | 24813 | 2053360 | 27543 | 82.75 | 74.55 |
| 19 | 8 | 10 | 1 | 22373 | 1817154 | 25224 | 81.22 | 72.04 |
| 20 | 9 | 10 | 1 | 9146 | 615340.1 | 10369 | 67.28 | 59.34 |
| 21 | 9 | 11 | 1 | 2949 | 174645.9 | 3255 | 59.22 | 53.65 |
| 22 | 9 | 12 | -15 | 7500 | 417026.1 | 8723 | 55.6 | 47.81 |
| 23 | 9 | 14 | -15 | 9171 | 642640.8 | 11356 | 70.07 | 56.59 |
| 24 | 10 | 14 | 1 | 9293 | 897499.5 | 12097 | 96.58 | 74.19 |
| 25 | 11 | 12 | 1 | 1020 | 57091.16 | 1211 | 55.97 | 47.14 |
| 26 | 11 | 13 | 1 | 6291 | 383950.1 | 6824 | 61.03 | 56.26 |
| 27 | 12 | 13 | -15 | 2877 | 158850.9 | 3161 | 55.21 | 50.25 |
| 28 | 12 | 14 | 1 | 35 | 3993.995 | 720 | 114.11 | 55.47 |
| 29 | 13 | 14 | 1 | 544 | 39699.01 | 686 | 72.98 | 57.87 |
| 30 | 13 | 15 | 1 | 372 | 28783.48 | 457 | 77.37 | 62.98 |
| 31 | 14 | 15 | 1 | 96 | 5008.929 | 132 | 52.18 | 37.95 |

**Table S6.** Characteristics of the mapped linkages between the 15 core areas for Himalayan goral in the CHAL. Here, lcDist= Least-cost distance, lcpLength= Least-cost path length, eucDist= Euclidean distance, cwd= cost weight distance

| Link | Core1 | Core 2 | linkType | eucDist | lcDist | lcpLength | *cwd:EucD* | *cwd:LCP* |
| --- | --- | --- | --- | --- | --- | --- | --- | --- |
| 1 | 1 | 2 | 1 | 783 | 38531.45 | 1189 | 49.21 | 32.41 |
| 2 | 1 | 4 | 1 | 3680 | 255298.2 | 4062 | 69.37 | 62.85 |
| 3 | 1 | 5 | -15 | 8061 | 506699.2 | 10926 | 62.86 | 46.38 |
| 4 | 2 | 3 | 1 | 3300 | 182604.5 | 3767 | 55.33 | 48.47 |
| 5 | 2 | 4 | 1 | 3616 | 204313.3 | 4592 | 56.5 | 44.49 |
| 6 | 3 | 4 | -15 | 9983 | 732338.2 | 17710 | 73.36 | 41.35 |
| 7 | 3 | 7 | 1 | 23270 | 1846458 | 27701 | 79.35 | 66.66 |
| 8 | 3 | 8 | -15 | 28402 | 2233098 | 35117 | 78.62 | 63.59 |
| 9 | 4 | 5 | 1 | 91 | 8603.417 | 144 | 94.54 | 59.75 |
| 10 | 4 | 6 | -15 | 3401 | 155075.4 | 4071 | 45.6 | 38.09 |
| 11 | 4 | 7 | 1 | 16885 | 1173078 | 19648 | 69.47 | 59.7 |
| 12 | 5 | 6 | 1 | 973 | 53291.75 | 1330 | 54.77 | 40.07 |
| 13 | 6 | 7 | -15 | 24921 | 1698124 | 36242 | 68.14 | 46.86 |
| 14 | 6 | 10 | -15 | 52053 | 4061832 | 71309 | 78.03 | 56.96 |
| 15 | 7 | 8 | 1 | 837 | 68486.05 | 929 | 81.82 | 73.72 |
| 16 | 7 | 10 | -15 | 25444 | 2046336 | 28940 | 80.43 | 70.71 |
| 17 | 8 | 9 | 1 | 24813 | 1828066 | 26566 | 73.67 | 68.81 |
| 18 | 8 | 10 | 1 | 22373 | 1788818 | 25693 | 79.95 | 69.62 |
| 19 | 9 | 10 | 1 | 9146 | 647761.3 | 10423 | 70.82 | 62.15 |
| 20 | 9 | 11 | 1 | 2949 | 187374.1 | 3193 | 63.54 | 58.68 |
| 21 | 9 | 12 | 1 | 7500 | 531883.9 | 8326 | 70.92 | 63.88 |
| 22 | 11 | 12 | 1 | 1020 | 105081.7 | 1087 | 103.02 | 96.67 |
| 23 | 12 | 14 | 1 | 35 | 4114.688 | 72 | 117.56 | 57.15 |
| 24 | 13 | 14 | 1 | 544 | 56023.75 | 600 | 102.98 | 93.37 |
| 25 | 13 | 15 | 1 | 372 | 39752.84 | 414 | 106.86 | 96.02 |
| 26 | 14 | 15 | 1 | 96 | 12729.55 | 132 | 132.6 | 96.44 |

**Table S7.** Characteristics of the mapped linkages between the 15 core areas for rhesus macaque in the CHAL. Here, lcDist= Least-cost distance, lcpLength= Least-cost path length, eucDist= Euclidean distance, cwd= cost weight distance

| Link | Core1 | Core 2 | linkType | eucDist | lcDist | lcpLength | *cwd:EucD* | *cwd:LCP* |
| --- | --- | --- | --- | --- | --- | --- | --- | --- |
| 1 | 1 | 2 | 1 | 783 | 47873.4 | 919 | 61.14 | 52.09 |
| 2 | 1 | 4 | 1 | 3680 | 158247.8 | 4209 | 43 | 37.6 |
| 3 | 1 | 5 | -15 | 8061 | 273200 | 18077 | 33.89 | 15.11 |
| 4 | 2 | 3 | 1 | 3300 | 218869.4 | 5472 | 66.32 | 40 |
| 5 | 2 | 4 | 1 | 3616 | 115907 | 4876 | 32.05 | 23.77 |
| 6 | 3 | 4 | 1 | 9983 | 551730.4 | 11970 | 55.27 | 46.09 |
| 7 | 3 | 7 | 1 | 23270 | 887661.4 | 30446 | 38.15 | 29.16 |
| 8 | 3 | 8 | 1 | 28402 | 1177293 | 37454 | 41.45 | 31.43 |
| 9 | 4 | 5 | 1 | 91 | 10292.98 | 144 | 113.11 | 71.48 |
| 10 | 4 | 6 | -15 | 3401 | 142553.1 | 6196 | 41.92 | 23.01 |
| 11 | 4 | 7 | 1 | 16885 | 415718.7 | 21126 | 24.62 | 19.68 |
| 12 | 5 | 6 | 1 | 973 | 36166.2 | 2168 | 37.17 | 16.68 |
| 13 | 6 | 7 | -15 | 24921 | 771801.7 | 40838 | 30.97 | 18.9 |
| 14 | 6 | 10 | -15 | 52053 | 2709824 | 77065 | 52.06 | 35.16 |
| 15 | 7 | 8 | 1 | 837 | 79698.84 | 941 | 95.22 | 84.7 |
| 16 | 7 | 10 | 1 | 25444 | 1659882 | 29380 | 65.24 | 56.5 |
| 17 | 8 | 9 | 1 | 24813 | 1858745 | 28252 | 74.91 | 65.79 |
| 18 | 8 | 10 | 1 | 22373 | 1564598 | 26862 | 69.93 | 58.25 |
| 19 | 9 | 10 | 1 | 9146 | 480740.1 | 10291 | 52.56 | 46.71 |
| 20 | 9 | 11 | 1 | 2949 | 198849.1 | 3427 | 67.43 | 58.02 |
| 21 | 9 | 12 | 1 | 7500 | 459018.6 | 8809 | 61.2 | 52.11 |
| 22 | 9 | 14 | -15 | 9171 | 580952.9 | 11775 | 63.35 | 49.34 |
| 23 | 10 | 14 | 1 | 9293 | 802913.1 | 11755 | 86.4 | 68.3 |
| 24 | 11 | 12 | 1 | 1020 | 52138.62 | 1156 | 51.12 | 45.1 |
| 25 | 11 | 13 | 1 | 6291 | 418122.4 | 7471 | 66.46 | 55.97 |
| 26 | 12 | 13 | -15 | 2877 | 189243.3 | 3143 | 65.78 | 60.21 |
| 27 | 12 | 14 | 1 | 35 | 3465.896 | 72 | 99.03 | 48.14 |
| 28 | 13 | 14 | 1 | 544 | 57498.72 | 600 | 105.7 | 95.83 |
| 29 | 13 | 15 | 1 | 372 | 29197.78 | 427 | 78.49 | 68.38 |
| 30 | 14 | 15 | 1 | 96 | 6202.955 | 132 | 64.61 | 46.99 |

**Table S8.** Characteristics of the mapped linkages between the 15 core areas for langur in the CHAL. Here, lcDist= Least-cost distance, lcpLength= Least-cost path length, eucDist= Euclidean distance, cwd= cost weight distance

| Link | Core1 | Core 2 | linkType | eucDist | lcDist | lcpLength | *cwd:EucD* | *cwd:LCP* |
| --- | --- | --- | --- | --- | --- | --- | --- | --- |
| 1 | 1 | 2 | 1 | 783 | 66392.99 | 834 | 84.79 | 79.61 |
| 2 | 1 | 4 | 1 | 3680 | 258449.4 | 4160 | 70.23 | 62.13 |
| 3 | 1 | 5 | 1 | 8061 | 569846 | 9943 | 70.69 | 57.31 |
| 4 | 2 | 3 | 1 | 3300 | 233614 | 3643 | 70.79 | 64.13 |
| 5 | 2 | 4 | 1 | 3616 | 258115.1 | 3878 | 71.38 | 66.56 |
| 6 | 3 | 4 | 1 | 9983 | 680863.2 | 10751 | 68.2 | 63.33 |
| 7 | 3 | 7 | 1 | 23270 | 1511510 | 27811 | 64.96 | 54.35 |
| 8 | 3 | 8 | 1 | 28402 | 1790663 | 34313 | 63.05 | 52.19 |
| 9 | 4 | 5 | 1 | 91 | 9460.867 | 144 | 103.97 | 65.7 |
| 10 | 4 | 6 | -15 | 3401 | 223633.3 | 4088 | 65.76 | 54.7 |
| 11 | 4 | 7 | 1 | 16885 | 919236.2 | 18604 | 54.44 | 49.41 |
| 12 | 5 | 6 | 1 | 973 | 83049.77 | 1131 | 85.35 | 73.43 |
| 13 | 6 | 7 | 1 | 24921 | 1471913 | 28014 | 59.06 | 52.54 |
| 14 | 6 | 10 | 1 | 52053 | 3147698 | 60341 | 60.47 | 52.17 |
| 15 | 7 | 8 | 1 | 837 | 65920.62 | 929 | 78.76 | 70.96 |
| 16 | 7 | 10 | -15 | 25444 | 1456580 | 32833 | 57.25 | 44.36 |
| 17 | 8 | 9 | 1 | 24813 | 1220034 | 27223 | 49.17 | 44.82 |
| 18 | 8 | 10 | 1 | 22373 | 1221544 | 26756 | 54.6 | 45.65 |
| 19 | 9 | 10 | 1 | 9146 | 494336.3 | 11010 | 54.05 | 44.9 |
| 20 | 9 | 11 | 1 | 2949 | 160457.6 | 3293 | 54.41 | 48.73 |
| 21 | 9 | 12 | -15 | 7500 | 417949.9 | 9065 | 55.73 | 46.11 |
| 22 | 9 | 13 | 1 | 9171 | 421193.2 | 10510 | 45.93 | 40.08 |
| 23 | 10 | 13 | 1 | 9293 | 611438.8 | 12739 | 65.8 | 48 |
| 24 | 12 | 14 | 1 | 1020 | 83503.16 | 1087 | 81.87 | 76.82 |
| 25 | 13 | 15 | 1 | 6291 | 391163.5 | 7147 | 62.18 | 54.73 |
| 26 | 12 | 15 | -15 | 2877 | 172626.6 | 3349 | 60 | 51.55 |
| 27 | 12 | 13 | 1 | 35 | 5450.112 | 72 | 155.72 | 75.7 |
| 28 | 11 | 12 | 1 | 544 | 46499.39 | 600 | 85.48 | 77.5 |

**Table S9.** Characteristics of the mapped linkages between the 15 core areas for Himalayan black bear in the CHAL. Here, lcDist= Least-cost distance, lcpLength= Least-cost path length, eucDist= Euclidean distance, cwd= cost weight distance

| Link | Core1 | Core 2 | linkType | eucDist | lcDist | lcpLength | *cwd:EucD* | *cwd:LCP* |
| --- | --- | --- | --- | --- | --- | --- | --- | --- |
| 1 | 1 | 2 | 1 | 783 | 38080.55 | 1493 | 48.63 | 25.51 |
| 2 | 1 | 4 | 1 | 3680 | 308209.9 | 3739 | 83.75 | 82.43 |
| 3 | 1 | 5 | -15 | 8061 | 682907.8 | 10096 | 84.72 | 67.64 |
| 4 | 2 | 3 | 1 | 3300 | 217181.2 | 3643 | 65.81 | 59.62 |
| 5 | 2 | 4 | 1 | 3616 | 252185.2 | 4149 | 69.74 | 60.78 |
| 6 | 3 | 4 | -15 | 9983 | 862029.9 | 16875 | 86.35 | 51.08 |
| 7 | 3 | 7 | 1 | 23270 | 2177344 | 26902 | 93.57 | 80.94 |
| 8 | 3 | 8 | 1 | 28402 | 2687813 | 33251 | 94.63 | 80.83 |
| 9 | 4 | 5 | 1 | 91 | 11203.06 | 144 | 123.11 | 77.8 |
| 10 | 4 | 6 | -15 | 3401 | 223805.4 | 3880 | 65.81 | 57.68 |
| 11 | 4 | 7 | 1 | 16885 | 1454478 | 19472 | 86.14 | 74.7 |
| 12 | 5 | 6 | 1 | 973 | 86899.26 | 1131 | 89.31 | 76.83 |
| 13 | 6 | 7 | -15 | 24921 | 2174618 | 31826 | 87.26 | 68.33 |
| 14 | 6 | 10 | -15 | 52053 | 5019964 | 60778 | 96.44 | 82.6 |
| 15 | 7 | 8 | 1 | 837 | 90463.55 | 923 | 108.08 | 98.01 |
| 16 | 7 | 10 | 1 | 25444 | 2408867 | 26974 | 94.67 | 89.3 |
| 17 | 8 | 9 | 1 | 24813 | 2147058 | 26021 | 86.53 | 82.51 |
| 18 | 8 | 10 | 1 | 22373 | 2121844 | 24241 | 94.84 | 87.53 |
| 19 | 9 | 10 | 1 | 9146 | 856722.7 | 10021 | 93.67 | 85.49 |
| 20 | 9 | 11 | 1 | 2949 | 264811.5 | 3094 | 89.8 | 85.59 |
| 21 | 9 | 12 | 1 | 7500 | 715717.3 | 8101 | 95.43 | 88.35 |
| 22 | 9 | 14 | 1 | 8071 | 872973.1 | 9859 | 108.16 | 82.36 |
| 23 | 10 | 14 | 1 | 9293 | 1092810 | 11547 | 117.59 | 94.64 |
| 24 | 11 | 12 | 1 | 1020 | 108451 | 1087 | 106.32 | 99.77 |
| 25 | 11 | 13 | 1 | 6291 | 640380.7 | 6764 | 101.79 | 94.67 |
| 26 | 11 | 14 | -15 | 4423 | 443295.4 | 4787 | 100.22 | 92.6 |
| 27 | 12 | 13 | -15 | 2277 | 284716.3 | 3008 | 125.04 | 94.65 |
| 28 | 12 | 14 | 1 | 35 | 6559.071 | 72 | 187.41 | 91.1 |
| 29 | 13 | 14 | 1 | 544 | 59927.07 | 600 | 110.16 | 99.88 |
| 30 | 13 | 15 | 1 | 372 | 41445.63 | 414 | 111.41 | 100.11 |
| 31 | 14 | 15 | 1 | 96 | 13213.15 | 132 | 137.64 | 100.1 |

**Table S10.** Characteristics of the mapped linkages between the 15 core areas for different mammals in the CHAL. Here, lcDist= Least-cost distance, lcpLength= Least-cost path length, eucDist= Euclidean distance, cwd= cost weight distance

| Link | Core1 | Core 2 | linkType | eucDist | lcDist | lcpLength | *cwd:EucD* | *cwd:LCP* |
| --- | --- | --- | --- | --- | --- | --- | --- | --- |
| 1 | 1 | 2 | 1 | 783 | 61030.29 | 1152 | 77.94 | 52.98 |
| 2 | 1 | 4 | 1 | 3680 | 227083.1 | 3888 | 61.71 | 58.41 |
| 3 | 1 | 5 | -15 | 8061 | 492808.1 | 10372 | 61.13 | 47.51 |
| 4 | 2 | 3 | 1 | 3300 | 230258.1 | 3643 | 69.78 | 63.21 |
| 5 | 2 | 4 | 1 | 3616 | 211705.9 | 4178 | 58.55 | 50.67 |
| 6 | 3 | 4 | 1 | 9983 | 692847.4 | 11208 | 69.4 | 61.82 |
| 7 | 3 | 7 | 1 | 23270 | 1585873 | 28579 | 68.15 | 55.49 |
| 8 | 3 | 8 | 1 | 28402 | 1884028 | 35192 | 66.33 | 53.54 |
| 9 | 4 | 5 | 1 | 91 | 8391.948 | 144 | 92.22 | 58.28 |
| 10 | 4 | 6 | -15 | 3401 | 186697.6 | 3993 | 54.89 | 46.76 |
| 11 | 4 | 7 | 1 | 16885 | 956745.4 | 19096 | 56.66 | 50.1 |
| 12 | 5 | 6 | 1 | 973 | 72382.9 | 1131 | 74.39 | 64 |
| 13 | 6 | 7 | 1 | 24921 | 1514266 | 27920 | 60.76 | 54.24 |
| 14 | 6 | 10 | 1 | 52053 | 3463198 | 60630 | 66.53 | 57.12 |
| 15 | 7 | 8 | 1 | 837 | 67924.85 | 929 | 81.15 | 73.12 |
| 16 | 7 | 10 | -15 | 25444 | 1744081 | 28938 | 68.55 | 60.27 |
| 17 | 8 | 9 | 1 | 24813 | 1614928 | 27055 | 65.08 | 59.69 |
| 18 | 8 | 10 | 1 | 22373 | 1554220 | 25837 | 69.47 | 60.15 |
| 19 | 9 | 10 | 1 | 9146 | 492394.8 | 10965 | 53.84 | 44.91 |
| 20 | 9 | 11 | 1 | 2949 | 149495.3 | 3168 | 50.69 | 47.19 |
| 21 | 9 | 12 | -15 | 7500 | 389392.8 | 9182 | 51.92 | 42.41 |
| 22 | 9 | 14 | 1 | 9171 | 482962.2 | 10507 | 52.66 | 45.97 |
| 23 | 10 | 14 | 1 | 9293 | 643004.9 | 12442 | 69.19 | 51.68 |
| 24 | 11 | 12 | 1 | 1020 | 76337.41 | 1099 | 74.84 | 69.46 |
| 25 | 11 | 13 | 1 | 6291 | 358752.7 | 7122 | 57.03 | 50.37 |
| 26 | 11 | 14 | 1 | 4423 | 245913.5 | 5229 | 55.6 | 47.03 |
| 27 | 12 | 13 | -15 | 2877 | 147515.7 | 3388 | 51.27 | 43.54 |
| 28 | 12 | 14 | 1 | 35 | 3692.243 | 72 | 105.49 | 51.28 |
| 29 | 13 | 14 | 1 | 544 | 38833.35 | 637 | 71.38 | 60.96 |
| 30 | 13 | 15 | 1 | 372 | 31922.78 | 414 | 85.81 | 77.11 |
| 31 | 14 | 15 | 1 | 96 | 8311.631 | 132 | 86.58 | 62.97 |
