# Supplementary material for: Landscape‐level habitat connectivity of large mammals in Chitwan Annapurna Landscape, Nepal
Source: Ecol Evol. 2024 Aug 16;14(8):e70087. doi: 10.1002/ece3.70087 (PMC11327774; doi:10.1002/ece3.70087)
Supplement: Supplementary file 2 — Data S2: [file ECE3-14-e70087-s001.docx]

**Supplementary information 2**

**Potential corridors for selected mammals**

In total, 31 linkages were identified on the basis of LCP values among 15 distinct habitat patches for the northern red muntjac. The matrix of linkages displayed variability among pairs of habitat patches. Notably, the CWD and EucD ratio was the lowest reached (CWD: EucD = 41.87) between patch two (referred to as Australian camp area) and patch three (known as Lumle area), signifying the presence of the highest-quality corridor between these patches. (Table 3a, Figure 2(a), Table S2).

Thirty different linkages were identified between the patches for chital. Relatively lower ratio of CWD and EucD was estimated between Devghat (14) and Barandabhar (15) (CWD:EucD = 83.74), but there was very weak relation of chital to other patches ( CWD:EucD>100) (Table 3b , Figure 2(b), Table S3).

The sambar was restricted to the lower region of the designated study area, the CHAL. The LCP analysis pointed a total of 32 linkages between habitat patches. However, the analysis revealed that nearly all the connections among the patches were weak (where CWD:EucD>100) and indicated a limited the movement of sambar between the patches across the landscape (Table 3c, Figure 3(a), Table S4).

This study identified 31 linkages between the patches for wild pigs. Among them, most of the linkages were characterized by low resistance (CWD: EucD<100) i.e., had low LCP except Panchase to Pipaltari (CWD: EucD = 132.22) and Ghumane to Kota area (CWD: EucD = 114.11) (Table 3d, Figure 3(b), Table S5). But the lowest LCP length was between the patches Ghumane to Kota area (LCP = 720 m).

There are 26 linkages between the patches for Himalayan goral. CWD and EucD ratio was 45.6, 49.21 between the patches 4 to 6 and 1 to 2 respectively, hence, had low resistance and high connectivity. But high resistance was seen in the patches in the low elevations (Table 3e, Figure 4, Table S6).

Thirty linkages were identified for monkeys. The LCP analysis revealed that majority of the patch pairs were favorable for monkeys (CWD: EucD<100). The result revealed the rhesus had low resistance in the LCP between the patches. The highest resistance for rhesus was seen in patches 4 to 5 and 13 to 14 (Table 3f, Figure 5(a), Table S7).

The outcomes revealed a similar pattern in patch connectivity for langurs as observed connection in rhesus macaques. The majority of the patches were suitable for langur movement and permitted easy travel through the LCP (CWD: EucD<100). However, relatively greater resistance was observed between patch pairs 12 and 13 (CWD: EucD = 155.72) as well as patches 4 and 5 (CWD: EucD = 103.97) (Table 3g, Figure 5(b), Table S8).

In the case of the Himalayan black bear, a total of 31 connections were seen among 15 significant habitat patches. The strong linkage was detected between patches 1 and 2, with a CWD:EucD ratio of 48.63, emphasized a strong association between these specific patches (Table 3h, Figure 6(a), Table S9). Likewise, the findings indicated weak linkages with patches located at lower elevations, where the CWD:EucD ratio was greater than 100.

A total of 31 linkages were identified for leopard. LCP analysis found that most of the habitat patches were interlinked with low resistances for leopards. That means identified habitat patches were suitable for leopard and had lower LCP (CWD: EucD>100) between patches. The range between the minimum and maximum CWD: EucD was 50.69 and 105.49 respectively (Table 3i, Figure 6(b), Table S10).
